# Optimized Potting Media and Vertical Farming Enhance High‐Value Crop Productivity in Riverine Lands of Bangladesh

**DOI:** 10.1002/pei3.70197

**Published:** 2026-07-31

**Authors:** Reema Ashrafi, Md. Selim Reza, Md. Parvez Kabir, Falguni Akter Shraboni, Md. Imran Hossain Sazib, Faridul Alam, Akbar Hossain

**Affiliations:** ^1^ Soil Science Division Bangladesh Institute of Nuclear Agriculture Mymensingh Bangladesh; ^2^ Soil Science Division Bangladesh Wheat and Maize Research Institute Dinajpur Bangladesh; ^3^ Wheat Breeding Division Bangladesh Wheat and Maize Research Institute Dinajpur Bangladesh; ^4^ Seed Section‐1, Seed Wing, Ministry of Agriculture Bangladesh Secretariat Dhaka Bangladesh; ^5^ Soils Unit Bangladesh Agricultural Research Council Dhaka Bangladesh

**Keywords:** char lands, high‐value crops, potting media, sustainable agriculture, vermicompost, vertical farming

## Abstract

Limited cultivable land and seasonal flooding constrain vegetable production in the riverine char lands of Bangladesh, highlighting the need for efficient and climate‐resilient production systems. This study evaluated optimized potting media for seedling production and the vertical cultivation of high‐value vegetables, particularly capsicum and spinach, as well as the integration of outdoor vertical farming with conventional farming to improve vegetable productivity and farm income. The results indicated that vermicompost:soil:sand (1:1:1) produced the most vigorous tomato and cauliflower seedlings, recording stem lengths of 10.30 and 4.32 cm, root lengths of 4.03 and 5.12 cm, leaf areas of 9.70 and 5.92 cm^2^, stem diameters of 2.10 and 1.30 mm, and fresh weights of 0.80 and 0.75 g seedling^−1^, respectively. For crop production, vermicompost:rice husk at 70:30 produced the highest spinach yield (349.9 g pot^−1^) and capsicum yield (509.0 g pot^−1^). Compared with conventional farming, which produced 111.7 kg garden^−1^, BDT 4515 gross return, BDT 3465 net return, and a benefit–cost ratio of 4.30, the integrated vertical‐conventional system produced 307.56 kg garden^−1^, BDT 10,083.30 gross return, BDT 7998.30 net return, and a benefit–cost ratio of 4.84. These findings indicate that locally available organic substrates, particularly vermicompost‐based mixtures, can improve vegetable productivity, farm income, and land‐use efficiency in flood‐prone riverine areas. Vertical farming may therefore offer a practical climate‐resilient option for enhancing food security and livelihoods among resource‐poor char land farmers.

## 1 Introduction

Food security is one of the most pressing challenges currently facing the world, especially in regions where available land for agriculture is scarce, and environmental stress, climate variability and resource deficiency hinder its productivity (Abebaw 2025; Saleem et al. 2025). This is particularly true of Bangladesh, due to its population density, its susceptibility to flooding and reliance on smallholder agriculture. The country is a place of high rainfall with the estimated mean annual rainfall of 2174 mm for the period 1991–2020, which varies from place to place (Hossain 2025). During the monsoon season, most of the rainfall is experienced, causing the possibility of floods, waterlogging and damage of crops in low lying farming lands.

The char lands are riverine sand bars created by the deposition of sediments and are home to almost 6.5 million people in Bangladesh (Alam and Khan 2022; Brammer 2021). The occupation of char lands is highly risky as low‐quality agricultural inputs, unstable soil condition, and flooding and erosion problems frequently affect these land types, especially agricultural production (Jahan et al. 2026; Rahman et al. 2025; Sarker et al. 2022; Karim et al. 2017). These limitations affect crop yields and household incomes, especially during the rainy season when crop cultivation is a challenge in the traditional system. Excess moisture and poor drainage can also have a negative impact on seed germination and seedling establishment, particularly when locally sourced substrates do not offer adequate water holding capacity, aeration, and nutrient supply (Zhou et al. 2020; Manik et al. 2019; Choudhary and Machavaram 2023).

Potting media are crucial to the quality of the seedlings and crop performance, affecting root growth, available moisture, nutrient availability, and aeration (Mir et al. 2022; Roy et al. 2024). Locally available materials like cocopeat, rice husk, sand, organic amendments, and soil are commonly used by farmers for making growing media. But these mixtures are typically chosen based on the availability of the material and not tested experimentally for performance (Jahan et al. 2024). Many substrates used in the local context, therefore, may not be conducive to good seedling vigor or crop yield in the vertical farming context, especially in flood‐prone char land environment.

As urbanization and climate change exert their challenges, traditional agriculture is no longer able to satisfy the food demands of the rising population (Misra and Ghosh 2024). Crop cultivation in vertical farms has the potential to be a method of enhancing vegetable production in regions where land is scarce or unavailable for a certain season. By using vertical space, stacked containers, trellises, fences, and homestead structures, this system can increase crop density and improve land‐use efficiency (Akintuyi 2024; Oh and Lu 2023; Jadhav et al. 2025). In riverine char lands, vertical farming may be particularly useful because it can reduce dependence on flood‐affected ground‐level cultivation and allow farmers to produce high‐value vegetables within limited homestead areas. However, the success of such systems depends strongly on the selection of suitable potting media that can sustain healthy seedlings and productive crop growth.

Despite growing interest in vertical farming, limited information is available on optimized substrate combinations for vertical vegetable production under the riverine conditions of Bangladesh. In particular, there is insufficient evidence on how locally available materials such as vermicompost, rice husk, cocopeat, soil, and sand perform in vertically stacked seedling systems and high‐value crop production (Rajiv and Kumari 2023). Moreover, few studies have evaluated whether outdoor vertical farming integrated with conventional homestead farming can improve total vegetable yield and economic returns for resource‐poor char land households (Lakhiar et al. 2025).

Therefore, this study tested the hypothesis that optimized potting media based on locally available organic substrates can improve seedling vigor, crop yield, and economic returns in vertical farming systems in riverine char lands. The specific objectives were to: (i) identify suitable potting media for raising tomato and cauliflower seedlings in vertically stacked layers; (ii) determine appropriate growing media for vertical cultivation of capsicum and spinach; and (iii) evaluate the productivity and profitability of outdoor vertical farming integrated with conventional homestead farming compared with conventional farming alone.

## 2 Materials and Methods

### 2.1 Experimental Site and Study Conditions

The study was conducted during the period of November 2022 to June 2023 at the homestead farms in Sadar, Mymensingh, Bangladesh to identify suitable potting media for seedling production and high‐value vegetable cultivation in vertical farming systems integrated with conventional farming. The experiments were carried out under outdoor homestead conditions using vertically stacked structures and conventional garden plots. The study included three experiments: (i) identification of suitable potting media for tomato and cauliflower seedling production; (ii) evaluation of growing media for vertical cultivation of capsicum and spinach; and (iii) assessment of outdoor vertical farming integrated with conventional farming in comparison with conventional farming alone.

#### 2.1.1 Weather Conditions During the Experimental Period

**FIGURE 1:**
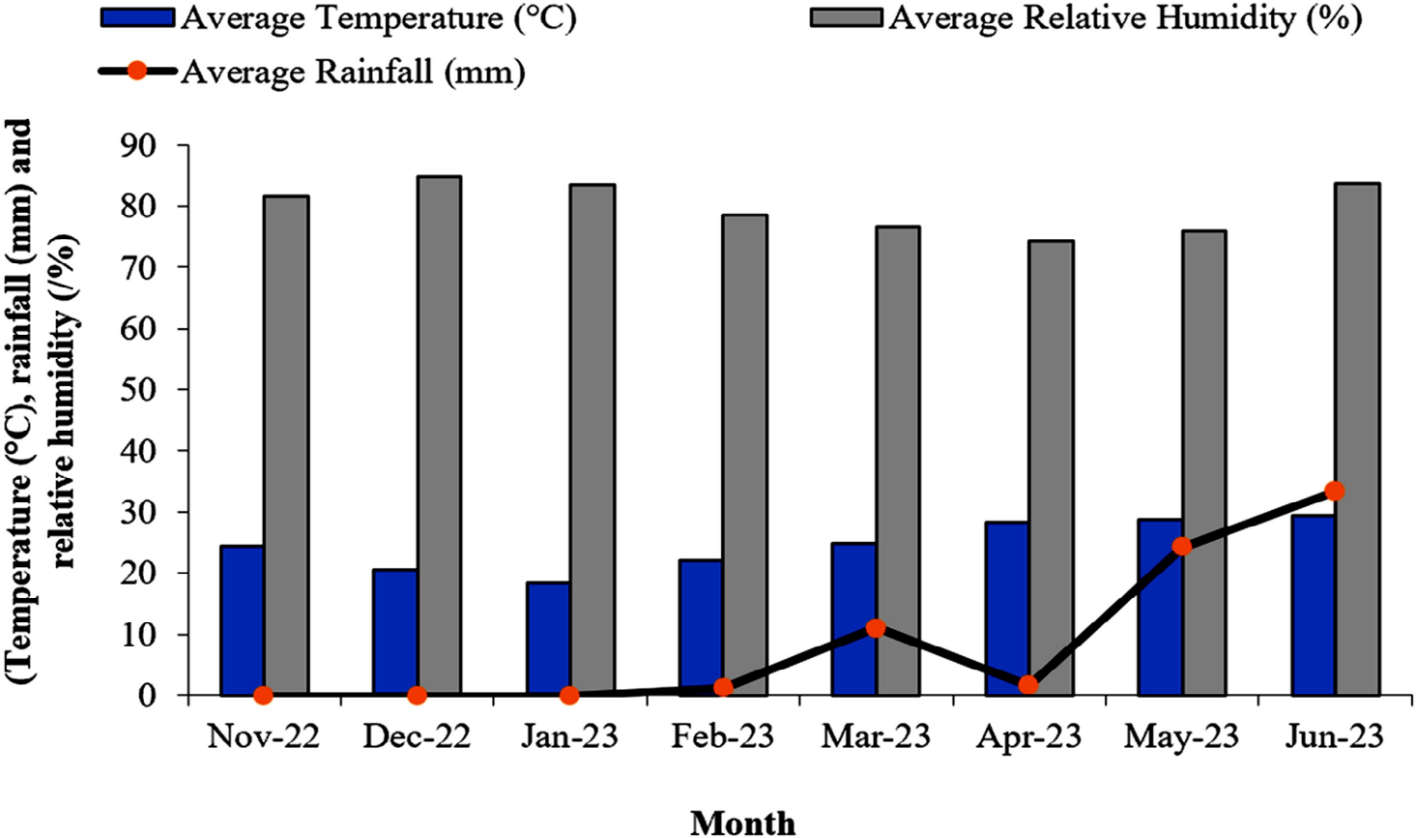
Monthly average temperature (°C), rainfall (mm), and relative humidity (%) during the experimental period from November 2022 to June 2023 at Sadar, Mymensingh, Bangladesh.

During the experimental period from November 2022 to June 2023, the monthly average temperature ranged from 18.4°C to 29.5°C, rainfall ranged from 0 to 33.4 mm, and relative humidity ranged from 74.2% to 84.8% (Figure 1).

The lowest temperature was recorded in January 2023, whereas the highest temperature and rainfall were recorded in June 2023. No rainfall was observed from November 2022 to January 2023. The monthly variation in temperature, rainfall, and relative humidity is presented in Figure 1.

### 2.2 Identification of Suitable Potting Media for Vertical Seedling Production

#### 2.2.1 Selection of Crop Varieties

Tomato ( *Solanum lycopersicum* L.) cv. Roma VF and cauliflower ( *Brassica oleracea* var. *botrytis* L.) cv. Atria F_1_ were selected for seedling production because of their high yield potential, popularity among local growers, and strong market demand.

#### 2.2.2 Preparation and Analysis of Potting Media

The potting media were prepared from locally available materials like soil, sand, vermicompost, cocopeat, and rice husk in various proportions as per treatments. Media were thoroughly mixed prior to filling seedling trays. The physical properties were measured such as bulk density, porosity, and water‐holding capacity. Chemical properties such as pH, electrical conductivity (EC), organic carbon (OC), nitrogen (N), phosphorus (P), potassium (K), sulfur (S), and carbon‐to‐nitrogen ratio were determined by following standard procedures.

#### 2.2.3 Experimental Design and Treatments

The experiment was laid out in a randomized complete block design (RCBD) with 10 treatments and three replications. The treatments were: T_1_: Soil:sand = 2:1, T_2_: Vermicompost = 100%, T_3_: Cocopeat = 100%, T_4_: Vermicompost:cocopeat = 2:1, T_5_: Vermicompost:cocopeat = 1:1, T_6_: Vermicompost:rice husk = 2:1, T_7_: Vermicompost:rice husk = 1:1, T_8_: Vermicompost:rice husk:cocopeat = 2:1:1, T_9_: Vermicompost:rice husk:cocopeat = 1:1:1 and T_10_: Vermicompost:soil:sand = 1:1:1.

#### 2.2.4 Seedling Production and Data Collection

**FIGURE 2:**
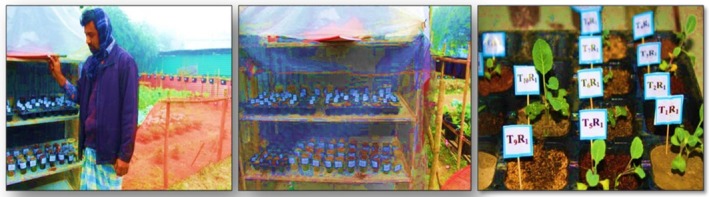
Vertical seedling nursery setup.

Seeds were sown in seed starter trays arranged in vertically stacked layers under outdoor homestead conditions (Figure 2). Seedlings were grown for 3–4 weeks, and data were collected on germination percentage, stem length, root length, number of leaves, leaf area, stem diameter, and fresh weight.

The germination percentage was calculated as follows:

$$\text{Germination}\;\left(\%\right)=\left(\frac{\text{Number of Germinated Seeds}}{\text{Total Seed Sown}}\right)\times 100$$

Stem length was measured from the seedling base to the shoot tip using a ruler and expressed in centimeters. Root length was measured from the stem base to the root tip and expressed in centimeters. The number of leaves per seedling was counted manually. Leaf area was measured using a LI‐3000C Portable Area Meter equipped with an LI‐3050C Transparent Belt Conveyor (LI‐COR Biosciences, Lincoln, NE, USA), and the results were expressed in cm^2^. Stem diameter was measured at the seedling base using a screw gauge and expressed in millimeters. Fresh weight was recorded immediately after harvest using a digital balance and expressed as g seedling^−1^.

### 2.3 Evaluation of Growing Media for Vertical Cultivation of Capsicum and Spinach

#### 2.3.1 Selection of Crop Varieties

Capsicum ( *Capsicum annuum* L.) cv. Sweet Beauty F_1_ and spinach ( *Spinacia oleracea* L.) cv. Saathi were selected to evaluate the suitability of different growing media for high‐value vegetable production in vertical farming systems.

#### 2.3.2 Preparation and Analysis of Growing Media

The growing media were prepared from cocopeat, vermicompost, rice husk, and soil according to the treatment combinations. Physical and chemical properties of the media were analyzed using the same procedures described in Section 2.2.2. The prepared media were placed into plastic pots/containers used in the vertical farming structure.

#### 2.3.3 Experimental Design and Treatments

The experiment was conducted in an RCBD with 15 treatments and three replications. The treatments were: T_1_: Cocopeat = 100%, T_2_: Vermicompost = 100%, T_3_: Cocopeat:rice husk = 70:30, T_4_: Vermicompost:rice husk = 70:30, T_5_: Cocopeat:rice husk:vermicompost = 30:20:50, T_6_: Cocopeat:rice husk:vermicompost = 30:30:40, T_7_: Cocopeat:rice husk:vermicompost = 30:40:30, T_8_: Cocopeat:rice husk:vermicompost = 40:20:40, T_9_: Cocopeat:rice husk:vermicompost = 40:30:30, T_10_: Cocopeat:rice husk:vermicompost = 40:40:20, T_11_: Cocopeat:rice husk:vermicompost = 50:20:30, T_12_: Cocopeat:rice husk:vermicompost = 50:30:20, T_13_: Cocopeat:rice husk:vermicompost = 50:40:10, T_14_: Cocopeat:rice husk:vermicompost = 60:20:20 and T_15_: Soil:cocopeat:vermicompost = 50:25:25.

#### 2.3.4 Crop Establishment, Management, and Data Collection

**FIGURE 3:**
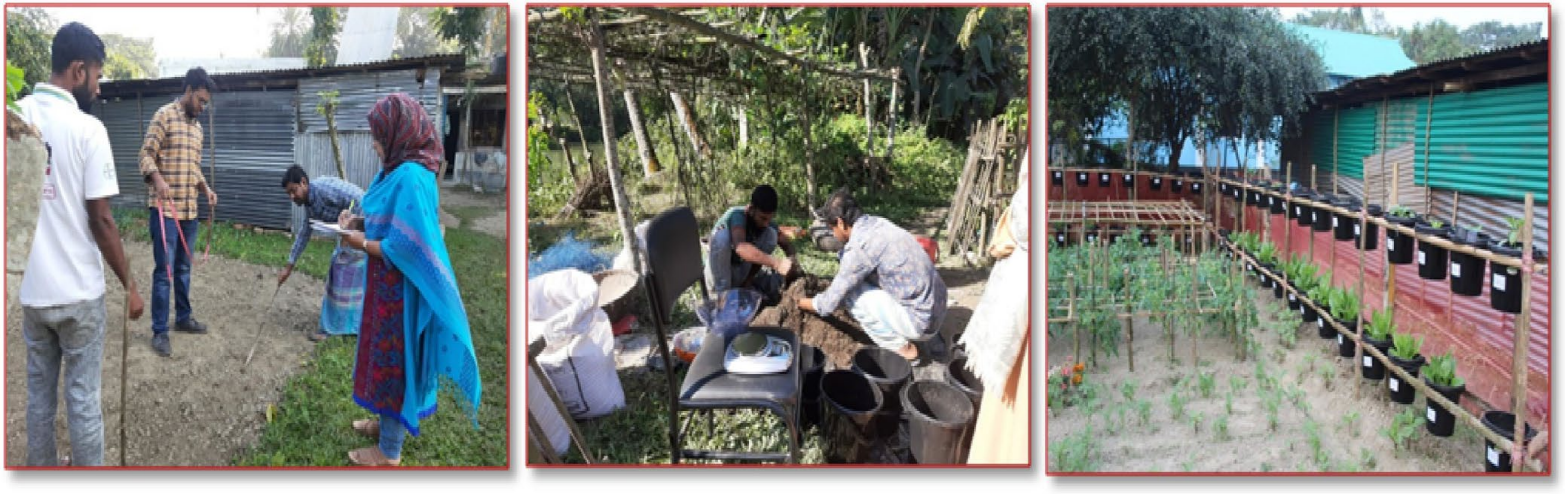
Vertical arrangement of containers for growing capsicum and spinach in the homestead garden setup.

The pots were arranged vertically in the homestead garden (Figure 3), using plastic containers measuring 25 cm in height and 15 cm in diameter. Five capsicum seeds were sown in each pot, whereas one spinach seedling was transplanted into each plastic pot. Irrigation was applied as needed to maintain adequate moisture in the growing media. Fertilizers were applied based on soil and substrate test results, following the recommended dose for each specific crop. Growth and yield parameters were recorded at harvest. This included plant height, number of leaves, number of fruits per pot for capsicum, and yield per pot for both capsicum and spinach (Figure 3).

### 2.4 Outdoor Vertical Farming Integrated With Conventional Farming

#### 2.4.1 Selection of Crops

Tomato, brinjal, carrot, broccoli, wax gourd, red amaranth, spinach, capsicum, and cowpea were selected for cultivation. These crops were chosen because of their suitability for homestead production, compatibility with vertical or conventional farming units, and high market value.

#### 2.4.2 Garden Layout and Crop Allocation

**FIGURE 4:**
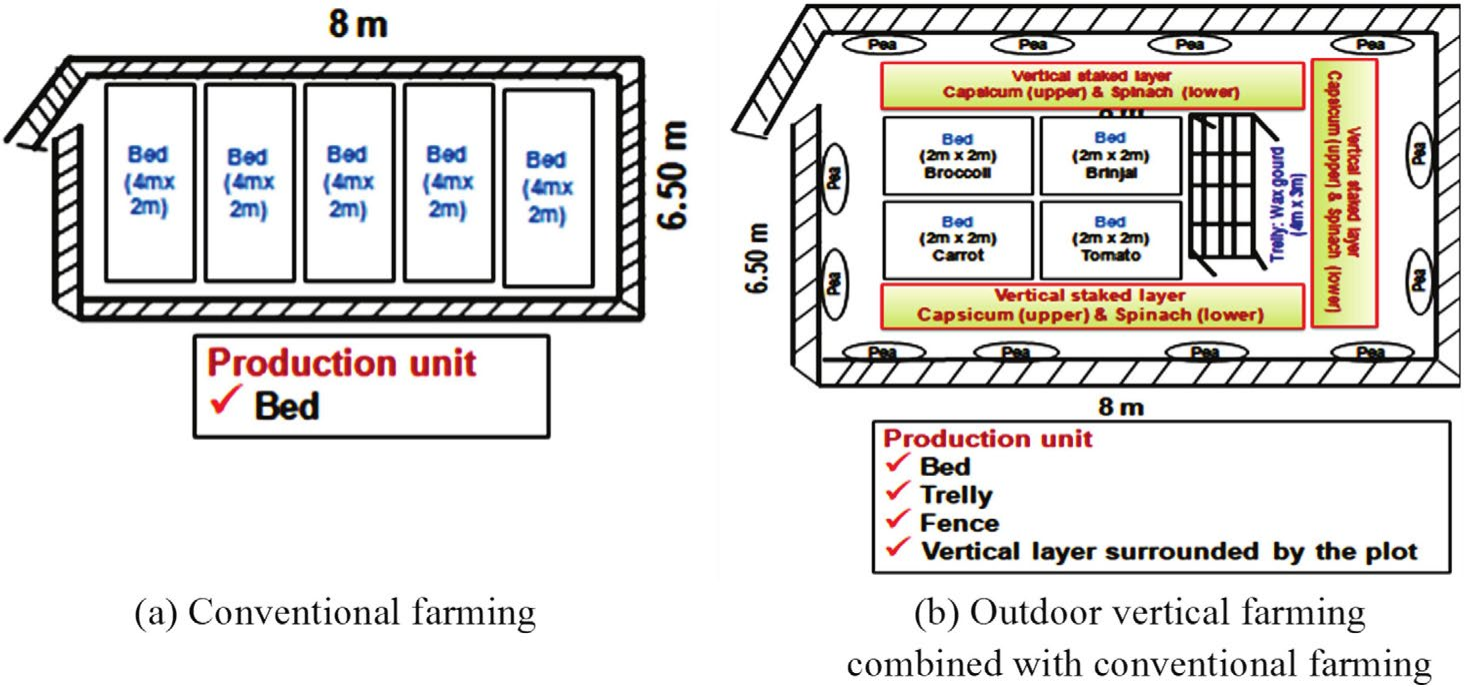
Layouts of (a) conventional farming and (b) outdoor vertical farming combined with conventional farming in the homestead garden.

**FIGURE 5:**
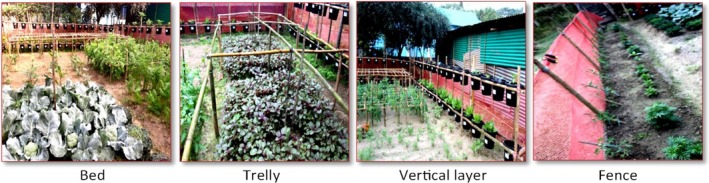
Crops were grown in various production units: (a) in beds, (b) on trellises, (c) in vertical layers and in fence.

An 8 m × 6.5 m homestead garden area was used to compare conventional farming alone with outdoor vertical farming integrated with conventional farming, as shown in Figure 4. The garden area was divided into distinct production units: beds, trellises, vertical layers, and fences (Figure 5).

Crops were allocated to each unit as follows:

- Beds: Cauliflower, carrot, tomato, and brinjal.
- Trellis: Wax gourd, with red amaranth planted beneath for the first month.
- Vertical Layers (containers stacked vertically): Spinach and capsicum.
- Fence: Cowpea.

This arrangement was designed to improve space use efficiency and allow different crops to grow in suitable production units within the same homestead area.

#### 2.4.3 Yield and Economic Analysis

Yield data were collected from each production unit and expressed as kg unit^−1^ or kg garden^−1^, as appropriate. Production costs and gross returns were recorded for both systems. Net return was calculated by subtracting total production cost from gross return. The benefit–cost ratio (BCR) was calculated as follows:

$$\mathrm{BCR}=\left(\frac{\text{Gross return}}{\text{Total production cost}}\right)$$

The economic performance of outdoor vertical farming integrated with conventional farming was then compared with conventional farming alone.

### 2.5 Statistical Analysis

Data from the first and second experiments were analyzed using analysis of variance (ANOVA) in M‐STAT‐C software. Because each experiment evaluated one factor, namely potting media composition, a one‐way ANOVA was performed separately for each crop and measured parameter. Treatment means were separated using Duncan's multiple range test (DMRT) at the 5% level of significance. Economic data from the third experiment were analyzed descriptively using total cost, gross return, net return, and BCR.

## 3 Results

The results are presented according to the three experimental objectives: potting media for seedling production, growing media for vertical cultivation of capsicum and spinach, and productivity and profitability of outdoor vertical farming integrated with conventional farming. To avoid repetition, treatment descriptions are provided in the table footnotes and are not repeatedly redefined in the text.

### 3.1 Identification of Suitable Potting Media for Vertical Seedling Production

#### 3.1.1 Physical Properties of Potting Media

The physical properties of the potting media differed markedly among treatments (Table 1 and Table S1).

**TABLE 1:** Physical properties of potting media for raising seedlings in vertically stacked layers.

Treatments	Bulk density (g/cm^3^)	Porosity (%)	Water holding capacity (%)
T_1_	0.89	55.22	30.86
T_2_	0.64	54.22	38.60
T_3_	0.11	67.57	56.38
T_4_	0.44	58.39	44.47
T_5_	0.40	60.14	46.13
T_6_	0.39	39.81	29.30
T_7_	0.31	36.15	22.24
T_8_	0.34	62.02	40.06
T_9_	0.29	62.61	34.58
T_10_	0.66	40.31	28.23
Range	0.11–0.89	36.15–67.57	22.24–56.38

*Note:* T_1_: Soil:sand = 2:1; T_2_: Vermicompost = 100%; T_3_: Cocopeat = 100%; T_4_: Vermicompost:cocopeat = 2:1; T_5_: Vermicompost:cocopeat = 1:1; T_6_: Vermicompost:rice husk = 2:1; T_7_: Vermicompost:rice husk = 1:1; T_8_: Vermicompost:rice husk:cocopeat = 2:1:1; T_9_: Vermicompost:rice husk:cocopeat = 1:1:1; and T_10_: Vermicompost:soil:sand = 1:1:1.

Bulk density ranged from 0.11 to 0.89 g cm^−3^, porosity from 36.15% to 67.57%, and water‐holding capacity from 22.24% to 56.38%. T_3_ had the lowest bulk density (0.11 g cm^−3^), highest water‐holding capacity (56.38%), and relatively high porosity (67.57%). Although T_3_ had favorable physical characteristics, T_10_ provided a better balance between physical structure and seedling performance in later growth assessments (Table 1 and Table S1).

#### 3.1.2 Chemical Properties of Potting Media

The chemical properties of the potting media varied across treatments (Table 2 and Table S2). The pH ranged from 6.35 to 8.22, indicating slightly acidic to alkaline conditions. Electrical conductivity ranged from 0.55 to 3.28, with T_2_ showing the highest EC. T_2_ also had the highest nitrogen (2.63%), phosphorus (0.4%), and sulfur (0.15%), whereas T_3_ had the highest organic carbon (36.86%) and potassium (1.08%) content. Although T_2_ was nutrient‐rich, T_10_ showed a more balanced nutrient profile and supported superior seedling growth.

**TABLE 2:** Chemical properties of potting media for raising seedlings in vertically stacked layers.

Treatments	pH	EC	%N	%P	%K	%S	%OC
T_1_	6.35	0.55	0.22	0.07	0.28	0.01	0.89
T_2_	6.70	3.28	2.63	0.4	0.13	0.15	21.17
T_3_	6.59	1.55	0.78	0.1	1.08	0.01	36.86
T_4_	6.78	2.05	1.85	0.4	0.74	0.11	24.02
T_5_	6.89	1.90	1.79	0.34	0.84	0.08	25.11
T_6_	8.22	1.93	1.68	0.34	0.76	0.05	35.24
T_7_	8.09	2.00	1.70	0.28	0.45	0.04	34.02
T_8_	7.46	1.66	2.02	0.39	0.73	0.09	30.07
T_9_	6.43	2.66	1.96	0.35	0.55	0.08	30.07
T_10_	6.73	1.73	0.89	0.13	0.36	0.02	04.68
Range	6.35–8.22	0.55–3.28	0.22–2.63	0.07–0.4	0.13–1.08	0.01–0.15	0.89–36.86

*Note:* Treatment details in Table 1.

#### 3.1.3 Growth Performance of Tomato Seedlings

Potting media significantly affected tomato seedling growth, except for germination percentage (Table 3 and Table S3). T_10_ produced the tallest seedlings, longest roots, largest leaf area, greatest stem diameter, and highest fresh weight. Specifically, T_10_ recorded 10.30 cm stem length, 4.03 cm root length, 9.70 cm^2^ leaf area, 2.10 mm stem diameter, and 0.80 g seedling^−1^ fresh weight. Therefore, T_10_ was identified as the best‐performing medium for tomato seedling production (Table 3 and Table S3).

**TABLE 3:** Effects of potting media on tomato seedling growth in vertically stacked layers.

Treatments	Germination (%)	Stem length (cm)	Root length (cm)	Leaves (no./seedling)	Leaf area (cm^2^)	Stem diameter (mm)	Fresh weight (g/seedling)
T_1_	100.00	6.37de	2.98cd	4.00a	6.28f	1.23e	0.47de
T_2_	66.67	5.67e	2.83d	4.33a	5.50g	1.53c	0.43e
T_3_	83.33	3.87f	2.08e	2.67b	1.73i	0.50f	0.26f
T_4_	100.00	7.10cd	3.10bc	4.33a	7.57d	1.43cd	0.57cd
T_5_	100.00	8.27b	3.84a	4.33a	8.77b	2.03ab	0.77ab
T_6_	83.33	0.78g	1.45f	4.00a	5.75g	1.17e	0.27f
T_7_	83.33	0.55g	1.06g	3.67a	2.76h	1.27de	0.27f
T_8_	100.00	7.46c	3.26b	4.33a	8.04c	1.90b	0.67bc
T_9_	66.67	0.73g	1.28fg	4.00a	7.09e	1.10e	0.37ef
T_10_	100.00	10.30a	4.03a	4.33a	9.70a	2.10a	0.80a
*F* test	NS	**	**	**	**	**	**
SE (±)	3.93	0.62	0.19	0.12	0.45	0.09	0.04
CV (%)	22.59	8.87	5.73	12.45	2.43	7	12.5

*Note:* Treatment details in Table 1. **, Significant at 1% level of probability. In a column, mean values having common letter(s) do not differ significantly, whereas mean values with dissimilar letter(s) differ significantly as per DMRT.

#### 3.1.4 Growth Performance of Cauliflower Seedlings

Cauliflower seedling growth also differed significantly among potting media, except for germination percentage (Table 4 and Table S4).

**TABLE 4:** Effects of potting media on cauliflower seedling growth in vertically stacked layers.

Treatment	Germination (%)	Stem length (cm)	Root length (cm)	Leaves (no./seedling)	Leaf area (cm^2^)	Stem diameter (mm)	Fresh weight (g/seedling)
T_1_	100.00	3.30cd	2.17e	4.33ab	4.81b	1.13abc	0.33bc
T_2_	66.67	2.63e	1.77fg	4.00abc	2.93c	1.00cd	0.22de
T_3_	83.33	3.40c	4.07c	2.33d	0.63g	1.08c	0.10f
T_4_	83.33	3.70b	4.58b	3.33c	4.89b	1.12bc	0.22de
T_5_	100.00	4.23a	3.30d	4.33ab	5.80a	1.28ab	0.38b
T_6_	100.00	2.52e	1.84f	4.00abc	2.20e	1.07cd	0.13ef
T_7_	66.67	2.33f	1.47h	3.33c	2.46d	1.10c	0.42b
T_8_	100.00	3.57b	1.63gh	3.67bc	0.95f	0.68e	0.13ef
T_9_	66.67	3.23d	1.85f	3.67bc	1.02f	0.90d	0.27cd
T_10_	100.00	4.32a	5.12a	4.67a	5.92a	1.30a	0.75a
*F*‐test	NS	**	**	**	**	**	**
SE (±)	4.11	0.12	0.24	0.14	0.36	0.04	0.03
CV (%)	23.02	2.88	4.2	12.93	3.62	9.53	16.92

*Note:* Treatment details in Table 1. **, Significant at 1% level of probability. In a column, mean values having common letter(s) do not differ significantly, whereas mean values with dissimilar letter(s) differ significantly as per DMRT.

T_10_ produced the strongest overall seedling performance, with the highest stem length, root length, number of leaves, leaf area, stem diameter, and fresh weight. T_10_ recorded 4.32 cm stem length, 5.12 cm root length, 4.67 leaves seedling^−1^, 5.92 cm^2^ leaf area, 1.30 mm stem diameter, and 0.75 g seedling^−1^ fresh weight. These results indicate that T_10_ was the most suitable potting medium for cauliflower seedling production (Table 4 and Table S4).

### 3.2 Evaluation of Growing Media for Vertical Cultivation of Capsicum and Spinach

#### 3.2.1 Physical Properties of Growing Media

The physical properties of the growing media used for capsicum and spinach varied among treatments (Table 5 and Table S5). Media weight ranged from 937.30 to 2499.10 g, bulk density from 0.11 to 0.64 g cm^−3^, water‐holding capacity from 17.96% to 64.35%, and porosity from 39.35% to 83.19%. T_14_ had the highest porosity, whereas T_11_ and T_14_ had the highest water‐holding capacity. However, physical properties alone did not determine yield, as T_4_ produced the highest crop yield despite having moderate values for bulk density (0.53 g cm^−3^), water‐holding capacity (19.31%), and porosity (48.54%) (Table 5 and Table S5).

**TABLE 5:** Physical properties of growing media used for vertical cultivation of capsicum and spinach.

Treatments	Media weight (dry basis) (g)	Bulk density (g/cm^3^)	Water holding capacity (%)	Porosity (%)
T_1_	937.30	0.11	56.38	67.57
T_2_	1603.96	0.64	38.60	54.22
T_3_	1194.56	0.56	51.81	79.86
T_4_	1752.87	0.53	19.31	48.54
T_5_	1322.81	0.47	33.75	64.19
T_6_	1268.42	0.49	21.24	42.76
T_7_	1459.87	0.35	17.96	39.35
T_8_	1379.29	0.46	34.81	55.68
T_9_	1416.06	0.52	37.22	63.40
T_10_	1548.54	0.40	25.48	43.71
T_11_	1467.32	0.21	64.35	79.94
T_12_	1394.88	0.19	52.34	71.35
T_13_	1447.49	0.18	50.83	72.63
T_14_	1568.60	0.19	64.35	83.19
T_15_	2499.10	0.47	50.23	68.42

*Note:* T_1_: Cocopeat = 100%; T_2_: Vermicompost = 100%; T_3_: Cocopeat:rice husk = 70:30; T_4_: Vermicompost:rice husk = 70:30; T_5_: Cocopeat:rice husk:vermicompost = 30:20:50; T_6_: Cocopeat:rice husk:vermicompost = 30:30:40; T_7_: Cocopeat:rice husk:vermicompost = 30:40:30; T_8_: Cocopeat:rice husk:vermicompost = 40:20:40; T_9_: Cocopeat:rice husk:vermicompost = 40:30:30; T_10_: Cocopeat:rice husk:vermicompost = 40:40:20; T_11_: Cocopeat:rice husk:vermicompost = 50:20:30; T_12_: Cocopeat:rice husk:vermicompost = 50:30:20; T_13_: Cocopeat:rice husk:vermicompost = 50:40:10; T_14_: Cocopeat:rice husk:vermicompost = 60:20:20; T_15_: Soil:cocopeat:vermicompost = 50:25:25.

#### 3.2.2 Chemical Properties of Growing Media

The chemical properties of the growing media are presented in Table 6 and Table S6.

**TABLE 6:** Chemical properties of growing media used for vertical cultivation of capsicum and spinach.

Treatments	pH	EC	%N	%P	%K	%S	%OC
T_1_	6.62	1.55	0.90	0.00	1.21	0.03	33.21
T_2_	6.78	3.32	2.63	0.38	0.70	0.16	19.54
T_3_	6.63	2.00	1.23	0.23	1.07	0.03	40.91
T_4_	6.70	2.13	1.79	0.40	0.76	0.10	28.35
T_5_	7.29	1.66	1.79	0.28	0.87	0.09	29.16
T_6_	7.21	1.63	1.62	0.29	0.81	0.04	35.24
T_7_	6.20	2.19	1.68	0.21	0.87	0.04	37.67
T_8_	7.05	1.65	1.68	0.28	0.87	0.04	33.21
T_9_	6.49	1.87	1.68	0.32	0.86	0.06	35.24
T_10_	6.21	2.05	1.57	0.25	0.82	0.03	36.05
T_11_	6.76	1.78	1.51	0.18	0.86	0.04	31.59
T_12_	6.46	1.88	1.51	0.23	0.95	0.04	36.86
T_13_	6.13	2.13	1.68	0.24	0.84	0.02	35.64
T_14_	6.10	1.68	1.68	0.18	0.95	0.09	28.35
T_15_	6.60	1.84	0.89	0.15	0.63	0.04	10.17

*Note:* Treatment details in Table 5.

The pH ranged from 6.10 to 7.29, whereas EC ranged from 1.55 to 3.32. T_2_ had the highest nitrogen (2.63%) and sulfur (0.16%) contents, and T_4_ had the highest phosphorus (0.40%) content. T_1_ contained the highest potassium (1.21%), whereas T_3_ had the highest organic carbon (40.91%) content. The nutrient profile of T_4_, particularly its relatively high phosphorus and adequate nitrogen supply, was associated with superior spinach and capsicum yield (Table 6 and Table S6).

#### 3.2.3 Yield of Spinach and Capsicum Under Vertical Farming

Growing media significantly affected spinach and capsicum yield as present in Table 7 and Table S7. T_4_ produced the highest spinach yield, 349.9 g pot^−1^, and the highest capsicum yield, 509.0 g pot^−1^. T_8_ also produced statistically high spinach yield (344.30 g pot^−1^) and the second‐highest capsicum yield (442.0 g pot^−1^). In contrast, T_1_ produced the lowest spinach (137.9 g pot^−1^) and capsicum (263 g pot^−1^) yield. Thus, T_4_ was the best‐performing growing medium for both high‐value crops under the vertical farming system (Table 7 and Table S7).

**TABLE 7:** Effects of growing media on spinach and capsicum yield under vertical farming.

Treatments	Spinach	Capsicum	
	Yield (g/pot)	Number (no./pot)	Yield (g/pot)
T_1_	137.9j	5.9e	263.0j
T_2_	253.9h	6.5de	297.7i
T_3_	306.9e	7.5abc	346.7e
T_4_	349.9a	7.8a	509.0a
T_5_	328.6bc	6.7cde	367.7d
T_6_	321.3cd	7.5ab	363.7d
T_7_	314.9de	6.9bcd	349.3e
T_8_	344.3a	7.9a	442.0b
T_9_	334.6b	7.1a‐d	404.3c
T_10_	331.3b	7.9a	398.0c
T_11_	272.9g	6.8bcd	322.7g
T_12_	286.3f	6.4de	327.0fg
T_13_	290.6f	6.6de	335.0f
T_14_	260.3h	4.8f	312.7h
T_15_	153.6i	4.5f	292.7i
*F* test	**	**	**
SE (±)	9.37	0.16	9.20
CV (%)	1.98	7.3	1.57

*Note:* Treatment details in Table 5. **, Significant at 1% level of probability. In a column, mean values having common letter(s) do not differ significantly, whereas mean values with dissimilar letter(s) differ significantly as per DMRT.

### 3.3 Productivity and Profitability of Outdoor Vertical Farming Integrated With Conventional Farming

#### 3.3.1 Vegetable Yield Under Integrated and Conventional Farming Systems

The integrated outdoor vertical farming system produced a higher total vegetable yield than conventional farming alone (Table 8 and Table S8). Conventional farming produced 111.7 kg garden^−1^, whereas outdoor vertical farming integrated with conventional farming produced 307.56 kg garden^−1^. The increase in yield was mainly due to additional production from trellis, vertical layer, and fence units, which allowed the cultivation of crops such as wax gourd, red amaranth, spinach, capsicum, and cowpea within the same homestead area (Table 8 and Table S8).

**TABLE 8:** Vegetable yield from conventional farming and outdoor vertical farming integrated with conventional farming.

Observation	Yield from bed (kg/unit)				Yield from trellis (kg/unit)		Yield from vertically staked layer (kg/unit)		Yield on fence (kg/unit)	Total yield (kg/garden)
Production unit (8 m × 6.5 m)	Tomato	Brinjal	Carrot	Broccoli	Wax gourd	Red amaranth	Spinach	Capsicum	Cowpea	
Conventional farming	0	35	37	32.7	0	0	0	0	0	111.7
Outdoor vertical farming combined with conventional farming	29.83	11.50	20.98	13.04	27.30	20.18	12.86	15.99	4.2	307.56

#### 3.3.2 Economic Performance of Integrated and Conventional Farming Systems

Outdoor vertical farming integrated with conventional farming generated higher economic returns than conventional farming alone (Table 9 and Table S8).

**TABLE 9:** Economic performance of conventional farming and outdoor vertical farming integrated with conventional farming.

Observation	Conventional farming				Outdoor vertical farming combined with conventional farming			
Production unit (8 m × 6.5 m)	Bed	Trellis	Vertical layer	Fence	Bed	Trellis	Vertical layer	Fence
Production (kg/production unit)	104.7	—	—	—	75.35	47.48	28.85	4.2
Gross return (BDT)	4515	—	—	—	3144.4	1353.20	5375.70	210
Total gross return (BDT)	4515				10083.30			
Total variable cost (BDT)	1050				2085			
Total net return (BDT)	3465				7998.3			
BCR	4.30				4.84			

*Note:* Price (BDT kg^−1^): Tomato‐40; Brinjal‐40; Carrot‐40, Broccoli‐50; Wax gourd‐20, Red Amaranth‐40; Spinach‐45, Capsicum‐300, Peas‐50.

Conventional farming produced a gross return of BDT 4515, net return of BDT 3465, and benefit–cost ratio of 4.30. In comparison, the integrated system produced a gross return of BDT 10,083.30, net return of BDT 7998.30, and benefit–cost ratio of 4.84. These results indicate that integrating vertical farming with conventional homestead gardening improved both productivity and profitability (Table 9 and Table S8).

## 4 Discussion

The present study demonstrated that vertical farming performance in riverine homestead systems depends on both substrate quality and efficient use of limited space. Across the seedling and crop‐production experiments, the best performance was achieved not by a single‐component medium, but by media that balanced aeration, water retention, nutrient availability, and root support. This agrees with previous studies showing that plastic container and soilless media must provide adequate water‐holding capacity, air‐filled porosity, nutrient buffering, and low physical resistance for root development (Gruda 2019; Caron and Michel 2021). In vertical systems, these properties are especially important because the root volume is restricted and moisture or nutrient imbalance can quickly affect plant growth.

### 4.1 Potting Media Effects on Tomato and Cauliflower Seedling Production

The physical properties of the seedling media strongly influenced root‐zone conditions. Cocopeat had low bulk density and high water‐holding capacity, which are commonly reported advantages of coconut coir‐based substrates (Mwesigwa 2017; Srinivas Naveen Kumar 2025). However, cocopeat alone did not produce the most vigorous seedlings, and it showed that good physical structure alone was insufficient when the supply of nutrients was low. Similar findings have been observed in container production, where cocopeat enhanced water retention and aeration but required nutrient enrichment to allow the maximum growth of plants (Chang et al. 2023).

Pure vermicompost and some vermicompost‐rice husk mixes contained higher levels of nutrients, but this did not always improve seedlings' growth. This may be due to greater electrical conductivity and less favorable air‐water balance. Vermicompost can boost seed germination, root growth, and seedling quality at appropriate proportions, but excessive amounts may increase soluble salts and limit early seedling growth (Evelin et al. 2019; Shabala and Munns 2017). Therefore, the response of tomato and cauliflower seedlings in the present study reveals that vermicompost should be mixed with materials that provide structural support, rather than being utilized alone.

The complementary nature of the components of vermicompost:soil:sand can be attributed to its superior performance at a ratio of 1:1:1. Vermicompost provided organic matter, nutrients, and biologically active substances; soil enhanced nutrient buffering, root anchorage; and sand enhanced drainage and decreased compaction. Combined substrate mixtures have been reported to enhance seedling morphology based on the combination of nutrient availability and appropriate physical structure (Pascual et al. 2018; Gruda 2019). In the present study, this balance resulted in greater stem length, root length, leaf area, stem diameter, and fresh weight in both tomato and cauliflower.

The stem diameter and fresh weight are significant signs of the quality of transplanting as they indicate mechanical strength, stored biomass, and the capacity for post‐transplant establishment. The stronger seedlings produced in vermicompost:soil:sand therefore indicate better transplant potential. These findings support the use of locally available balanced media for seedling production in char land homesteads, where farmers require low‐cost and reliable nursery systems during flood‐prone periods.

### 4.2 Growing Media Effects on Spinach and Capsicum Production

The yield of spinach and capsicum was also determined by the interaction between physical and chemical media properties. Media with high porosity and moderate water‐holding capacity can improve oxygen diffusion and moisture availability in vertical containers. However, the highest yield was not obtained from cocopeat alone, confirming that structural suitability must be supported by adequate nutrition.

The vermicompost:rice husk mixture at 70:30 produced the highest yield of both spinach and capsicum. This result may be attributed to nutrient enrichment from vermicompost and improved aeration from rice husk. Vermicompost supplies plant‐available nitrogen, phosphorus, potassium, sulfur, and organic carbon, whereas rice husk or rice husk‐derived substrates improve porosity and drainage in container media (Imtiaz 2022; Rai et al. 2024; Mupambwa and Mnkeni 2018; Walia and Kaur 2024). Previous soilless‐culture work also showed that combining vermicompost with rice husk biochar improved nutrient uptake and yield in leafy vegetables, supporting the trend observed in the present study (Nurhidayati et al. 2022).

The yield of spinach is closely related to the expansion of the leaves and availability of nutrients, particularly nitrogen, whereas capsicum yield depends on the availability of nutrients for vegetative growth, flowering, and fruit development. The strong performance of vermicompost:rice husk suggests that this medium provided a suitable balance of nutrient availability and root‐zone aeration. Soil‐based media did not yield as well as the other media, likely due to increased weight and bulk density of the soil, which limited root growth and oxygen in the vertical containers. These findings confirm that media for vertical vegetable production should be lightweight, porous, and nutritionally balanced.

### 4.3 Productivity and Profitability of Integrated Vertical Farming

The integration of outdoor vertical farming with conventional homestead farming substantially increased vegetable production. The integrated system generated 307.56 kg garden^−1^ while the conventional farming alone produced 111.7 kg garden^−1^. The rise was mainly attributable to the higher yield from trellises, vertical layers and fence cultivation. This resulted in crops such as spinach, capsicum, wax gourd, scarlet amaranth, cowpea being cultivated inside the same 8 m × 6.5 m household area. This outcome is in accordance with the main premise of vertical farming, that is, raising the crop density per unit land area and increasing the production by utilizing the vertical space. Recent reviews have pointed out that vertical farming can be used to boost land‐use efficiency and produce high‐value vegetables in space‐constrained areas (Sowmya et al. 2024; Gokulakrishnan et al. 2025). This strategy is of practical benefit for smallholder households in riverine char regions where cultivable ground is often restricted, unstable or flooded annually.

The integrated system also improved profitability. Conventional farming generated a net return of BDT 3465.00 with a benefit–cost ratio of 4.30, whereas the integrated system generated a net return of BDT 7998.30 with a benefit–cost ratio of 4.84. Although the integrated system required higher production costs, the additional investment was offset by greater yield and inclusion of high‐value crops such as capsicum. Therefore, vertical farming can improve both productivity and income from small homestead areas.

### 4.4 Sustainability, Climate Resilience, and Livelihood Implications

The findings have important implications for sustainable and climate‐resilient agriculture in riverine Bangladesh. Char lands are frequently affected by flooding, erosion, and seasonal land instability, which reduces the reliability of conventional ground‐level cultivation. Vertical farming can reduce dependence on flood‐affected land by shifting part of vegetable production to raised containers, trellises, and homestead structures.

The use of locally available substrates such as vermicompost, rice husk, soil, sand, and cocopeat also supports resource‐efficient production. Vermicompost improves nutrient recycling and organic matter use, whereas rice husk adds value to an agricultural by‐product. Such systems can reduce dependence on external inputs and improve the sustainability of small‐scale vegetable production. In addition, producing diverse vegetables such as spinach, capsicum, cowpea, and leafy crops can improve household dietary diversity, food security, and income stability.

### 4.5 Limitations and Future Research

This study offers valuable evidence regarding integrated vertical farming and potting media in homestead settings; however, it is important to recognize certain constraints. Experiments were conducted at one riverine site, and system performance may vary with seasons, soil types, flood severity, and management conditions. Also, several crop and substrate combinations were included in the study. Long‐term media reuse, fertilizer depletion, pest pressure, irrigation scheduling, labor requirements, and construction durability were not extensively investigated.

Future research should test these media and vertical farming systems across multiple char land locations and seasons. Further work should also evaluate water‐use efficiency, nutrient‐release patterns, media recycling potential, cost of vertical structures, farmer adoption, and long‐term profitability. Such studies would help refine low‐cost vertical farming models for wider use in flood‐prone and land‐scarce agricultural regions.

## 5 Conclusion

This study demonstrated that optimized potting media and integrated vertical farming can improve vegetable productivity and profitability in riverine homestead agriculture of Bangladesh. Vermicompost:soil:sand at 1:1:1 was the most suitable medium for producing vigorous tomato and cauliflower seedlings, whereas vermicompost:rice husk at 70:30 produced the highest spinach and capsicum yields under vertical cultivation. Integrating outdoor vertical farming with conventional farming increased total vegetable yield from 111.7 to 307.56 kg garden^−1^ and improved net return from BDT 3465 to BDT 7998.30. The benefit–cost ratio also increased from 4.30 to 4.84, indicating greater economic efficiency. These findings suggest that vertical farming using locally available organic substrates can improve land‐use efficiency, crop diversity, food security, and income generation for smallholder farmers in flood‐prone char land areas. Future research should evaluate this system across multiple seasons and locations including long‐term media reuse, irrigation management, labor requirements, and farmer adoption potential.

## Funding

The study was financially supported by National Science and Technology (NST) Fellowship, Ministry of Science and Technology (MoST), Bangladesh.

## Ethics Statement

The authors have nothing to report.

## Conflicts of Interest

The authors declare no conflicts of interest.

## Supporting information


**Table S1:** Physical properties of potting media (Expt. 1: Identification of suitable potting media for tomato and cauliflower seedling production).
**Table S2:** Chemical properties of potting media (Expt. 1: Identification of suitable potting media for tomato and cauliflower seedling production).
**Table S3:** Raw data recorded for tomato grown under plastic glass house (Expt. 1: Identification of suitable potting media for tomato and cauliflower seedling production).
**Table S4:** Raw data for cauliflower grown under plastic glass house (Expt. 1: Identification of suitable potting media for tomato and cauliflower seedling production).
**Table S5:** Physical properties of growing media/treatments (Expt. 2: Evaluation of growing media for vertical cultivation of capsicum and spinach).
**Table S6:** Chemical properties of growing media/treatments (Expt. 2: Evaluation of growing media for vertical cultivation of capsicum and spinach).
**Table S7:** Raw data recorded for yield and yield attributes of capsicum and spinach (Expt. 2: Evaluation of growing media for vertical cultivation of capsicum and spinach).
**Table S8:** Raw data for vegetable yield and profitability under integrated and conventional farming systems (Expt. 3: Productivity and profitability of outdoor vertical farming integrated with conventional farming).

## Data Availability

All data are available in Supporting Information and in tables and figures.

## References

[pei370197-bib-0001] Abebaw, S. E. 2025. “A Global Review of the Impacts of Climate Change and Variability on Agricultural Productivity and Farmers' Adaptation Strategies.” Food Science & Nutrition 13, no. 5: e70260. 10.1002/fsn3.70260. 40370417 PMC12076006

[pei370197-bib-0002] Akintuyi, O. B. 2024. “Vertical Farming in Urban Environments: A Review of Architectural Integration and Food Security.” Open Access Research Journal of Biology and Pharmacy 10, no. 2: 114–126. 10.53022/oarjbp.2024.10.2.0017.

[pei370197-bib-0003] Alam, A. K. , and M. S. H. Khan . 2022. “Geomorphology of Bangladesh and Potential Land Use.” In Bangladesh Geosciences and Resources Potential, 355–398. CRC Press. 10.1201/9781003080817-10.

[pei370197-bib-0004] Brammer, H. 2021. “The Soils of Charlands in Bangladesh.” In Living on the Edge: Char Dwellers in Bangladesh, 167–183. Springer International Publishing. 10.1007/978-3-030-73592-0_10.

[pei370197-bib-0005] Caron, J. , and J. C. Michel . 2021. “Understanding and Optimizing the Physical Properties of Growing Media for Soilless Cultivation.” In Advances in Horticultural Soilless Culture, 107–137. Burleigh Dodds Science Publishing. 10.1201/9781003048206-6.

[pei370197-bib-0006] Chang, Y. Y. , M. F. A. Razak , and C. C. Sim . 2023. “Effects of Different Growing Media Under Soilless Culture on the Growth and Nutrient Uptake of Oil Palm Seedlings in the Pre‐Nursery Stage.” Science & Technology Asia 28, no. 4: 256–263.

[pei370197-bib-0007] Choudhary, V. , and R. Machavaram . 2023. “A Comprehensive Review of Sustainable Soil Organic Growing Media for Mat‐Type Paddy Seedling Nurseries Under Indian Agronomical Condition.” Journal of Soil Science and Plant Nutrition 23, no. 2: 1515–1534. 10.1007/s42729-023-01153-2.

[pei370197-bib-0008] Evelin, H. , T. S. Devi , S. Gupta , and R. Kapoor . 2019. “Mitigation of Salinity Stress in Plants by Arbuscular Mycorrhizal Symbiosis: Current Understanding and New Challenges.” Frontiers in Plant Science 10: 470. 10.3389/fpls.2019.00470. 31031793 PMC6473083

[pei370197-bib-0009] Gokulakrishnan, G. , D. Tamilselvan , S. Sanjeevkumar , A. Devaganesh , N. Janani , and K. Arunadevi . 2025. “Vertical Farming: Innovations, Challenges, and the Future of Sustainable Urban Agriculture.” Madras Agricultural Journal 112, no. 10–12: 43–49. 10.29321/MAJ.10.901236.

[pei370197-bib-0010] Gruda, N. S. 2019. “Increasing Sustainability of Growing Media Constituents and Stand‐Alone Substrates in Soilless Culture Systems.” Agronomy 9, no. 6: 298. 10.3390/agronomy9060298.

[pei370197-bib-0011] Hossain, K. M. I. 2025. “Climate Change in Bangladesh: A Synthesis of Risks, Adaptation, and Policy Responses.” International Journal of Business and Social and Scientific Research 13, no. 1: 44–51. 10.55706/ijbssr13106.

[pei370197-bib-0012] Imtiaz, S. 2022. “Production of Tomato Using Soilless Planting Media on Rooftop.” Doctoral Dissertation, Department of Agroforestry and Environmental Science, Sher‐e‐Bangla Agricultural University, Dhaka 1207, Bangladesh.

[pei370197-bib-0013] Jadhav, V. , T. Grondona , A. Pistillo , et al. 2025. “Optimizing Planting Density for Increased Resource Use Efficiency in Baby‐Leaf Production of Lettuce ( *Lactuca sativa* L.) and Basil ( *Ocimum basilicum* L.) in Vertical Farms.” Horticulturae 11, no. 4: 343. 10.3390/horticulturae11040343.

[pei370197-bib-0014] Jahan, F. , J. E. T. Momo , N. C. Herbert , et al. 2026. “Adaptive Strategies to Extreme Climate Events Among Riverine Communities in Sirajganj, Bangladesh.” Discover Environment 4, no. 1: 33. 10.1007/s44274-026-00524-7.

[pei370197-bib-0015] Jahan, H. , M. W. Rahman , B. Banik , et al. 2024. “Comparative Cost and Return Analysis of Eight Major Vegetables in Char Land Ecosystem of Bangladesh.” SAARC Journal of Agriculture 22, no. 1: 223–234. 10.3329/sja.v22i1.69775.

[pei370197-bib-0016] Karim, M. A. , M. A. Quayyum , S. Samsuzzaman , H. Higuchi , and E. Nawata . 2017. “Challenges and Opportunities in Crop Production in Different Types of Char Lands of Bangladesh: Diversity in Crops and Cropping.” Tropical Agriculture and Development 61, no. 2: 77–93. 10.11248/jsta.61.77.

[pei370197-bib-0017] Lakhiar, I. A. , H. Yan , T. N. Syed , et al. 2025. “Soilless Agricultural Systems: Opportunities, Challenges, and Applications for Enhancing Horticultural Resilience to Climate Change and Urbanization.” Horticulturae 11, no. 6: 568. 10.3390/horticulturae11060568.

[pei370197-bib-0018] Manik, S. N. , G. Pengilley , G. Dean , B. Field , S. Shabala , and M. Zhou . 2019. “Soil and Crop Management Practices to Minimize the Impact of Waterlogging on Crop Productivity.” Frontiers in Plant Science 10: 140. 10.3389/fpls.2019.00140. 30809241 PMC6379354

[pei370197-bib-0019] Mir, M. S. , N. B. Naikoo , R. H. Kanth , et al. 2022. “Vertical Farming: The Future of Agriculture: A Review.” Pharma Innovation Journal 11, no. 2: 1175–1195.

[pei370197-bib-0020] Misra, S. , and A. Ghosh . 2024. “Agriculture Paradigm Shift: A Journey From Traditional to Modern Agriculture.” In Biodiversity and Bioeconomy, 113–141. Elsevier. 10.1016/B978-0-323-95482-2.00006-7.

[pei370197-bib-0021] Mupambwa, H. A. , and P. N. S. Mnkeni . 2018. “Optimizing the Vermicomposting of Organic Wastes Amended With Inorganic Materials for Production of Nutrient‐Rich Organic Fertilizers: A Review.” Environmental Science and Pollution Research 25, no. 11: 10577–10595. 10.1007/s11356-018-1328-4. 29480396

[pei370197-bib-0022] Mwesigwa, I. 2017. “Modifying the Pore Structure of Coco‐Peat as a Growing Medium for Horticultural Crops in Greenhouse.” Doctoral Dissertation, Busitema University.

[pei370197-bib-0023] Nurhidayati, N. , A. S. Ansari , A. Sholihah , and P. N. Chiangmai . 2022. “Vermicompost and Rice Husk Biochar Interaction Ameliorates Nutrient Uptake and Yield of Green Lettuce Under Soilless Culture.” Journal of Horticultural Research 30, no. 2: 55–66. 10.2478/johr-2022-0018.

[pei370197-bib-0024] Oh, S. , and C. Lu . 2023. “Vertical Farming‐Smart Urban Agriculture for Enhancing Resilience and Sustainability in Food Security.” Journal of Horticultural Science and Biotechnology 98, no. 2: 133–140. 10.1080/14620316.2022.2141666.

[pei370197-bib-0025] Pascual, J. A. , F. Ceglie , Y. Tuzel , et al. 2018. “Organic Substrate for Transplant Production in Organic Nurseries. A Review.” Agronomy for Sustainable Development 38, no. 3: 35. 10.1007/s13593-018-0508-4.

[pei370197-bib-0026] Rahman, M. A. , M. S. Alam , R. Sultana , and R. Sultana . 2025. “Assessing the Interrelationship Between Monsoon Flood Disasters and Major Crop Production in Bangladesh.” International Journal of Disaster Risk Reduction 121: 105401. 10.1016/j.ijdrr.2025.105401.

[pei370197-bib-0027] Rai, A. , S. Smriti , and S. Kaushal . 2024. “Utilising Crop Residues as Hydroponic Media for Sustainable Food Production System.” International Journal of Research in Agronomy 7, no. 4: 73–78. 10.33545/2618060X.2024.v7.i4b.528.

[pei370197-bib-0028] Rajiv , and M. Kumari . 2023. “Protected Cultivation of High‐Value Vegetable Crops Under Changing Climate.” In Advances in Research on Vegetable Production Under a Changing Climate, vol. 2, 229–266. Springer International Publishing. 10.1007/978-3-031-20840-9_11.

[pei370197-bib-0029] Roy, A. S. , S. Das , D. Saha , and S. Barat . 2024. “Vertical Farming: A Sustainable Agriculture Format of the Future.” International Journal of Research in Agronomy 7, no. 4S: 308–314. 10.33545/2618060X.2024.v7.i4Sd.641.

[pei370197-bib-0030] Saleem, A. , S. Anwar , T. Nawaz , et al. 2025. “Securing a Sustainable Future: The Climate Change Threat to Agriculture, Food Security, and Sustainable Development Goals.” Journal of Umm Al‐Qura University for Applied Sciences 11, no. 3: 595–611. 10.1007/s43994-024-00177-3.

[pei370197-bib-0031] Sarker, U. K. , M. S. Kaysar , M. R. Uddin , M. A. Hossain , S. Hassan , and M. M. Hassan . 2022. “Exploring Farmers' Insight on Cropping Pattern for Sustainable Crop Production in Char Area of Bangladesh.” Sustainability 14, no. 3: 1745. 10.3390/su14031745.

[pei370197-bib-0032] Shabala, S. , and R. Munns . 2017. “Salinity Stress: Physiological Constraints and Adaptive Mechanisms.” In Plant Stress Physiology, 24–63. Cabi. 10.1079/9781780647296.0024.

[pei370197-bib-0033] Sowmya, C. , M. Anand , C. Indu Rani , G. Amuthaselvi , and P. Janaki . 2024. “Recent Developments and Inventive Approaches in Vertical Farming.” Frontiers in Sustainable Food Systems 8: 1400787. 10.3389/fsufs.2024.1400787.

[pei370197-bib-0034] Srinivas Naveen Kumar, G. J. 2025. “Effect of Rooting Media and PGRs on Vegetative Propagation of African Marigold ( *Tagetes erecta* L.).” Master's Thesis, Rani Lakshmi Bai Central Agricultural University. College of Horticulture, Department of Floriculture and Landscaping.

[pei370197-bib-0035] Walia, S. S. , and T. Kaur . 2024. “Beneficial Role of Vermicompost: Nutrient Content in Vermicompost and Success Stories.” In Earthworms and Vermicomposting: Species, Procedures and Crop Application, 135–146. Springer Nature Singapore. 10.1007/978-981-99-8953-9_13.

[pei370197-bib-0036] Zhou, W. , F. Chen , Y. Meng , et al. 2020. “Plant Waterlogging/Flooding Stress Responses: From Seed Germination to Maturation.” Plant Physiology and Biochemistry 148: 228–236. 10.1016/j.plaphy.2020.01.020. 31981875

