# Supplementary material for: Optimized Potting Media and Vertical Farming Enhance High‐Value Crop Productivity in Riverine Lands of Bangladesh
Source: Plant Environ Interact. 2026 Jul 31;7(4):e70197. doi: 10.1002/pei3.70197 (PMC13425633; doi:10.1002/pei3.70197)
Supplement: Supplementary file 1 — Table S1: Physical properties of potting media (Expt. 1: Identification of suitable potting media for tomato and cauliflower seedling production). Table S2: Chemical properties of potting media (Expt. 1: Identification of suitable potting media for tomato and cauliflower seedling production). Table S3: Raw data recorded for tomato grown under plastic glass house (Expt. 1: Identification of suitable potting media for tomato and cauliflower seedling production). Table S4: Raw data for cauliflower grown under plastic glass house (Expt. 1: Identification of suitable potting media for tomato and cauliflower seedling production). Table S5: Physical properties of growing media/treatments (Expt. 2: Evaluation of growing media for vertical cultivation of capsicum and spinach). Table S6: Chemical properties of growing media/treatments (Expt. 2: Evaluation of growing media for vertical cultivation of capsicum and spinach). Table S7: Raw data recorded for yield and yield attributes of capsicum and spinach (Expt. 2: Evaluation of growing media for vertical cultivation of capsicum and spinach). Table S8: Raw data for vegetable yield and profitability under integrated and conventional farming systems (Expt. 3: Productivity and profitability of outdoor vertical farming integrated with conventional farming). [file PEI3-7-e70197-s001.docx]

**Table S1**. Physical properties of potting media (Expt. 1: Identification of suitable potting media for tomato and cauliflower seedling production)

| **Growing media/treatments** | **Soil+ Cocopeat weight** | **Cocopeat weight** | **Saturation** | **Field capacity** | **Dry weight** | **Volume**  **(cm^3^)** | **Initial weight** | **Saturation** | **Field capacity** | **Dry weight** | **Mass of water**  **(gm)** | **Pore volume**  **Cm^3^** | **Porosity** | **Mass of water for WHC (gm)** | **Volume of water**  **(cm^3^)** | **WHC (%)** | **Bulk density**  **gm/cm^3^** |
| --- | --- | --- | --- | --- | --- | --- | --- | --- | --- | --- | --- | --- | --- | --- | --- | --- | --- |
| Soil:Sand=2:1 | 105.51 | 48.54 | 135.42 | 120.73 | 102.12 | 60.30 | 56.97 | 86.88 | 72.19 | 53.58 | 33.30 | 33.30 | 55.22 | 18.61 | 18.61 | 30.86 | 0.89 |
| Vermicompost=100% | 202.35 | 143.73 | 235.04 | 223.01 | 193.29 | 77.00 | 58.62 | 91.31 | 79.28 | 49.56 | 41.75 | 41.75 | 54.22 | 29.72 | 29.72 | 38.60 | 0.64 |
| Cocopeat=100% | 117.66 | 94.38 | 156.48 | 147.69 | 103.40 | 78.56 | 23.28 | 62.10 | 53.31 | 9.02 | 53.08 | 53.08 | 67.57 | 44.29 | 44.29 | 56.38 | 0.11 |
| Vermicompost:Cocopeat=2:1 | 103.27 | 68.57 | 143.86 | 133.64 | 101.00 | 73.40 | 34.70 | 75.29 | 65.07 | 32.43 | 42.86 | 42.86 | 58.39 | 32.64 | 32.64 | 44.47 | 0.44 |
| Vermicompost:Cocopeat=1:1 | 179.17 | 146.28 | 223.09 | 212.30 | 176.78 | 77.00 | 32.89 | 76.81 | 66.02 | 30.50 | 46.31 | 46.31 | 60.14 | 35.52 | 35.52 | 46.13 | 0.40 |
| Vermicompost:Rice husk=2:1 | 153.55 | 115.43 | 176.66 | 168.52 | 145.81 | 77.50 | 38.12 | 61.23 | 53.09 | 30.38 | 30.85 | 30.85 | 39.81 | 22.71 | 22.71 | 29.30 | 0.39 |
| Vermicompost:Rice husk=1:1 | 126.77 | 94.38 | 147.18 | 136.25 | 118.78 | 78.56 | 32.39 | 52.80 | 41.87 | 24.40 | 28.40 | 28.40 | 36.15 | 17.47 | 17.47 | 22.24 | 0.31 |
| Vermicompost:Rice husk:Cocopeat=2:1:1 | 111.26 | 82.30 | 161.84 | 143.70 | 110.61 | 82.60 | 28.96 | 79.54 | 61.40 | 28.31 | 51.23 | 51.23 | 62.02 | 33.09 | 33.09 | 40.06 | 0.34 |
| Vermicompost:Rice husk:Cocopeat=1:1:1 | 111.18 | 84.51 | 149.85 | 129.93 | 105.36 | 71.06 | 26.67 | 65.34 | 45.42 | 20.85 | 44.49 | 44.49 | 62.61 | 24.57 | 24.57 | 34.58 | 0.29 |
| Vermicompost:Soil:Sand=1:1:1 | 162.63 | 84.41 | 195.85 | 180.74 | 153.77 | 104.40 | 78.22 | 111.44 | 96.33 | 69.36 | 42.08 | 42.08 | 40.31 | 26.97 | 29.47 | 28.23 | 0.66 |

**Table S2**. Chemical properties of potting media (Expt. 1: Identification of suitable potting media for tomato and cauliflower seedling production)

| **Growing media/treatments** | **pH** | **EC** | **%N** | **%P** | **%K** | **%S** | **%OC** |
| --- | --- | --- | --- | --- | --- | --- | --- |
| Soil:Sand=2:1 | 6.35 | 0.545 | 0.224 | 0.07 | 0.275 | 0.01 | 0.89 |
| Vermicompost=100% | 6.7 | 3.28 | 2.632 | 0.4 | 0.13 | 0.145 | 21.17 |
| Cocopeat=100% | 6.59 | 1.55 | 0.784 | 0.1 | 1.082 | 0.014 | 36.86 |
| Vermicompost:Cocopeat=2:1 | 6.78 | 2.05 | 1.848 | 0.4 | 0.742 | 0.107 | 24.02 |
| Vermicompost:Cocopeat=1:1 | 6.89 | 1.9 | 1.792 | 0.34 | 0.84 | 0.083 | 25.11 |
| Vermicompost:Rice husk=2:1 | 8.22 | 1.93 | 1.681 | 0.34 | 0.759 | 0.048 | 35.24 |
| Vermicompost:Rice husk=1:1 | 8.09 | 2 | 1.68 | 0.28 | 0.452 | 0.037 | 34.02 |
| Vermicompost:Rice husk:Cocopeat=2:1:1 | 7.46 | 1.66 | 2.016 | 0.39 | 0.729 | 0.089 | 30.07 |
| Vermicompost:Rice husk:Cocopeat=1:1:1 | 6.43 | 2.66 | 1.96 | 0.35 | 0.549 | 0.079 | 30.07 |
| Vermicompost:Soil:Sand=1:1:1 | 6.73 | 1.73 | 0.896 | 0.13 | 0.355 | 0.023 | 4.68 |

**Table S3.** Raw data recorded for tomato grown under plastic glass house (Expt. 1: Identification of suitable potting media for tomato and cauliflower seedling production)

| **Treatment** | **Germination%** | **Stem length**  **(cm)** | **Root length**  **(cm)** | **Number of leaves (no./seedling)** | **Leaf area**  **(cm^3^)** | **Stem diameter**  **(cm)** | **Fresh weight**  **(gm/seedling)** |
| --- | --- | --- | --- | --- | --- | --- | --- |
| T1R1 | 100.00 | 6.90 | 2.80 | 4.00 | 6.31 | 1.30 | 0.40 |
| T1R2 | 100.00 | 5.70 | 2.95 | 4.00 | 6.19 | 1.20 | 0.50 |
| T1R3 | 100.00 | 6.50 | 3.20 | 4.00 | 6.35 | 1.20 | 0.50 |
| **Mean** | **100.00** | **6.37** | **2.98** | **4.00** | **6.28** | **1.23** | **0.47** |
| T2R1 | 50.00 | 5.00 | 2.80 | 4.00 | 5.34 | 1.50 | 0.40 |
| T2R2 | 50.00 | 6.10 | 2.94 | 4.00 | 5.69 | 1.70 | 0.50 |
| T2R3 | 100.00 | 5.90 | 2.74 | 5.00 | 5.47 | 1.40 | 0.40 |
| **Mean** | **66.67** | **5.67** | **2.83** | **4.33** | **5.50** | **1.53** | **0.43** |
| T3R1 | 100.00 | 3.80 | 2.23 | 3.00 | 1.69 | 0.50 | 0.30 |
| T3R2 | 100.00 | 3.60 | 2.10 | 3.00 | 1.71 | 0.40 | 0.20 |
| T3R3 | 50.00 | 4.20 | 1.90 | 2.00 | 1.78 | 0.60 | 0.30 |
| **Mean** | **83.33** | **3.87** | **2.08** | **2.67** | **1.73** | **0.50** | **0.27** |
| T4R1 | 100.00 | 7.90 | 3.25 | 5.00 | 7.60 | 1.50 | 0.60 |
| T4R2 | 100.00 | 6.20 | 3.10 | 4.00 | 7.59 | 1.40 | 0.60 |
| T4R3 | 100.00 | 7.20 | 2.95 | 4.00 | 7.51 | 1.40 | 0.50 |
| **Mean** | **100.00** | **7.10** | **3.10** | **4.33** | **7.57** | **1.43** | **0.57** |
| T5R1 | 100.00 | 8.30 | 3.70 | 4.00 | 8.65 | 2.00 | 0.70 |
| T5R2 | 100.00 | 7.90 | 3.90 | 4.00 | 8.78 | 2.10 | 0.80 |
| T5R3 | 100.00 | 8.60 | 3.93 | 5.00 | 8.87 | 2.20 | 0.90 |
| **Mean** | **100.00** | **8.27** | **3.84** | **4.33** | **8.77** | **2.10** | **0.80** |
| T6R1 | 50.00 | 0.74 | 1.04 | 4.00 | 5.93 | 1.10 | 0.28 |
| T6R2 | 100.00 | 0.89 | 1.06 | 4.00 | 5.43 | 1.30 | 0.25 |
| T6R3 | 100.00 | 0.72 | 1.07 | 4.00 | 5.89 | 1.10 | 0.26 |
| **Mean** | **83.33** | **0.78** | **1.06** | **4.00** | **5.75** | **1.17** | **0.26** |
| T7R1 | 50.00 | 0.51 | 1.45 | 3.00 | 2.97 | 1.10 | 0.20 |
| T7R2 | 100.00 | 0.56 | 1.30 | 4.00 | 2.45 | 1.40 | 0.30 |
| T7R3 | 100.00 | 0.59 | 1.60 | 4.00 | 2.86 | 1.30 | 0.30 |
| **Mean** | **83.33** | **0.55** | **1.45** | **3.67** | **2.76** | **1.27** | **0.27** |
| T8R1 | 100.00 | 6.99 | 3.15 | 4.00 | 7.96 | 1.80 | 0.70 |
| T8R2 | 100.00 | 7.89 | 3.33 | 5.00 | 8.10 | 1.90 | 0.60 |
| T8R3 | 100.00 | 7.50 | 3.30 | 4.00 | 8.05 | 2.00 | 0.70 |
| **Mean** | **100.00** | **7.46** | **3.26** | **4.33** | **8.04** | **1.90** | **0.67** |
| T9R1 | 50.00 | 0.74 | 1.23 | 4.00 | 7.02 | 1.00 | 0.40 |
| T9R2 | 50.00 | 0.73 | 1.20 | 4.00 | 7.12 | 1.20 | 0.30 |
| T9R3 | 100.00 | 0.73 | 1.41 | 4.00 | 7.14 | 1.10 | 0.40 |
| **Mean** | **66.67** | **0.73** | **1.28** | **4.00** | **7.09** | **1.10** | **0.37** |
| T10R1 | 100.00 | 10.10 | 4.20 | 4.00 | 9.71 | 2.00 | 0.70 |
| T10R2 | 100.00 | 10.60 | 3.80 | 5.00 | 9.52 | 2.10 | 0.80 |
| T10R3 | 100.00 | 10.20 | 4.10 | 4.00 | 9.88 | 2.00 | 0.80 |
| **Mean** | **100.00** | **10.30** | **4.03** | **4.33** | **9.70** | **2.03** | **0.77** |

**Table S4.** Raw data for cauliflower grown under plastic glass house (Expt. 1: Identification of suitable potting media for tomato and cauliflower seedling production)

| **Treatment** | **Germination%** | **Stem length**  **(cm)** | **Root length**  **(cm)** | **Number of leaves (no./seedling))** | **Leaf area**  **(cm^3^)** | **Stem diameter**  **(mm)** | **Fresh weight**  **(gm/seedling)** |
| --- | --- | --- | --- | --- | --- | --- | --- |
| T1R1 | 100.00 | 3.40 | 2.20 | 4.00 | 4.92 | 1.00 | 0.30 |
| T1R2 | 100.00 | 3.20 | 2.30 | 4.00 | 4.81 | 1.25 | 0.30 |
| T1R3 | 100.00 | 3.30 | 2.00 | 5.00 | 4.69 | 1.15 | 0.40 |
| **Mean** | **100.00** | **3.30** | **2.17** | **4.33** | **4.81** | **1.13** | **0.33** |
| T2R1 | 50.00 | 2.80 | 1.90 | 4.00 | 3.12 | 0.90 | 0.25 |
| T2R2 | 50.00 | 2.60 | 1.60 | 4.00 | 2.86 | 1.00 | 0.20 |
| T2R3 | 100.00 | 2.50 | 1.80 | 4.00 | 2.81 | 1.10 | 0.20 |
| **Mean** | **66.67** | **2.63** | **1.77** | **4.00** | **2.93** | **1.00** | **0.22** |
| T3R1 | 100.00 | 3.40 | 5.20 | 2.00 | 0.61 | 1.00 | 0.10 |
| T3R2 | 50.00 | 3.30 | 5.00 | 2.00 | 0.68 | 1.10 | 0.10 |
| T3R3 | 100.00 | 3.50 | 5.15 | 3.00 | 0.59 | 1.15 | 0.10 |
| **Mean** | **83.33** | **3.40** | **5.12** | **2.33** | **0.63** | **1.08** | **0.10** |
| T4R1 | 50.00 | 3.80 | 4.50 | 3.00 | 5.09 | 1.10 | 0.20 |
| T4R2 | 100.00 | 3.70 | 4.63 | 3.00 | 4.80 | 1.25 | 0.20 |
| T4R3 | 100.00 | 3.60 | 4.60 | 4.00 | 4.78 | 1.00 | 0.25 |
| **Mean** | **83.33** | **3.70** | **4.58** | **3.33** | **4.89** | **1.12** | **0.22** |
| T5R1 | 100.00 | 4.20 | 3.20 | 4.00 | 5.85 | 1.30 | 0.40 |
| T5R2 | 100.00 | 4.10 | 3.50 | 5.00 | 5.76 | 1.25 | 0.30 |
| T5R3 | 100.00 | 4.40 | 3.20 | 4.00 | 5.80 | 1.30 | 0.45 |
| **Mean** | **100.00** | **4.23** | **3.30** | **4.33** | **5.80** | **1.28** | **0.38** |
| T6R1 | 100.00 | 2.40 | 1.82 | 4.00 | 2.37 | 1.20 | 0.10 |
| T6R2 | 100.00 | 2.30 | 1.90 | 4.00 | 1.95 | 1.10 | 0.20 |
| T6R3 | 100.00 | 2.30 | 1.80 | 4.00 | 2.29 | 0.90 | 0.10 |
| **Mean** | **100.00** | **2.33** | **1.84** | **4.00** | **2.20** | **1.07** | **0.13** |
| T7R1 | 50.00 | 2.40 | 1.40 | 3.00 | 2.45 | 1.00 | 0.80 |
| T7R2 | 100.00 | 2.50 | 1.50 | 4.00 | 2.57 | 1.20 | 0.70 |
| T7R3 | 50.00 | 2.65 | 1.50 | 3.00 | 2.36 | 1.10 | 0.75 |
| **Mean** | **66.67** | **2.52** | **1.47** | **3.33** | **2.46** | **1.10** | **0.75** |
| T8R1 | 100.00 | 3.60 | 1.70 | 3.00 | 0.98 | 0.60 | 0.10 |
| T8R2 | 100.00 | 3.50 | 1.60 | 4.00 | 0.96 | 0.80 | 0.20 |
| T8R3 | 100.00 | 3.60 | 1.60 | 4.00 | 0.92 | 0.65 | 0.10 |
| **Mean** | **100.00** | **3.57** | **1.63** | **3.67** | **0.95** | **0.68** | **0.13** |
| T9R1 | 50.00 | 3.30 | 1.80 | 4.00 | 1.12 | 1.00 | 0.20 |
| T9R2 | 50.00 | 3.15 | 1.75 | 4.00 | 0.98 | 0.90 | 0.30 |
| T9R3 | 100.00 | 3.25 | 2.00 | 3.00 | 0.95 | 0.80 | 0.30 |
| **Mean** | **66.67** | **3.23** | **1.85** | **3.67** | **1.02** | **0.90** | **0.27** |
| T10R1 | 100.00 | 4.40 | 4.10 | 4.00 | 5.80 | 1.30 | 0.40 |
| T10R2 | 100.00 | 4.30 | 4.00 | 5.00 | 6.00 | 1.24 | 0.40 |
| T10R3 | 100.00 | 4.25 | 4.10 | 5.00 | 5.95 | 1.37 | 0.45 |
| **Mean** | **100.00** | **4.32** | **4.07** | **4.67** | **5.92** | **1.30** | **0.42** |

**Table S5**. Physical properties of growing media/treatments (Expt. 2: Evaluation of growing media for vertical cultivation of capsicum and spinach)

| **Growing media/treatments** | **Soil+ Cocopeat weight** | **Cocopeat weight** | **Saturation** | **Field capacity** | **Dry weight** | **Volume**  **(cm^3^)** | **Initial weight** | **Saturation** | **Field capacity** | **Dry weight** | **Mass of water**  **(gm)** | **Pore volume**  **cm^3^** | **Porosity** | **Mass of water for WHC (gm)** | **Volume of water**  **(cm^3^)** | **WHC (%)** | **Bulk density**  **gm/cm^3^** |
| --- | --- | --- | --- | --- | --- | --- | --- | --- | --- | --- | --- | --- | --- | --- | --- | --- | --- |
| Cocopeat=100% | 117.66 | 94.38 | 156.48 | 147.69 | 103.40 | 78.56 | 23.28 | 62.10 | 53.31 | 9.02 | 53.08 | 53.08 | 67.57 | 44.29 | 44.29 | 56.38 | 0.11 |
| Vermicompost=100% | 202.35 | 143.73 | 235.04 | 223.01 | 193.29 | 77.00 | 58.62 | 91.31 | 79.28 | 49.56 | 41.75 | 41.75 | 54.22 | 29.72 | 29.72 | 38.60 | 0.64 |
| Cocopeat:Rice husk=70:30 | 100.00 | 72.21 | 164.54 | 145.45 | 110.18 | 68.07 | 27.79 | 92.33 | 73.24 | 37.97 | 54.36 | 54.36 | 79.86 | 35.27 | 35.27 | 51.81 | 0.56 |
| Vermicompost:Rice husk=70:30 | 104.49 | 68.57 | 133.29 | 114.69 | 102.40 | 63.64 | 35.92 | 64.72 | 46.12 | 33.83 | 30.89 | 30.89 | 48.54 | 12.29 | 12.29 | 19.31 | 0.53 |
| Cocopeat:Rice husk:Vermicompost=30:20:50 | 178.54 | 146.28 | 231.93 | 208.49 | 182.50 | 77.00 | 32.26 | 85.65 | 62.21 | 36.22 | 49.43 | 49.43 | 64.19 | 25.99 | 25.99 | 33.75 | 0.47 |
| Cocopeat:Rice husk:Vermicompost=30:30:40 | 150.62 | 115.43 | 186.26 | 169.58 | 153.12 | 77.50 | 35.19 | 70.83 | 54.15 | 37.69 | 33.14 | 33.14 | 42.76 | 16.46 | 16.46 | 21.24 | 0.49 |
| Cocopeat:Rice husk:Vermicompost=30:40:30 | 121.47 | 94.38 | 152.71 | 135.91 | 121.80 | 78.56 | 27.09 | 58.33 | 41.53 | 27.42 | 30.91 | 30.91 | 39.35 | 14.11 | 14.11 | 17.96 | 0.35 |
| Cocopeat:Rice husk:Vermicompost=40:20:40 | 114.44 | 82.30 | 166.38 | 149.14 | 120.39 | 82.60 | 32.14 | 84.08 | 66.84 | 38.09 | 45.99 | 45.99 | 55.68 | 28.75 | 28.75 | 34.81 | 0.46 |
| Cocopeat:Rice husk:Vermicompost=40:30:30 | 116.98 | 84.51 | 166.22 | 147.62 | 121.17 | 71.06 | 32.47 | 81.71 | 63.11 | 36.66 | 45.05 | 45.05 | 63.40 | 26.45 | 26.45 | 37.22 | 0.52 |
| Cocopeat:Rice husk:Vermicompost=40:40:20 | 116.46 | 84.41 | 171.72 | 152.69 | 126.09 | 104.40 | 32.05 | 87.31 | 68.28 | 41.68 | 45.63 | 45.63 | 43.71 | 26.60 | 26.60 | 25.48 | 0.40 |
| Cocopeat:Rice husk:Vermicompost=50:20:30 | 79.13 | 48.54 | 120.16 | 109.04 | 63.16 | 71.30 | 30.59 | 71.62 | 60.50 | 14.62 | 57.00 | 57.00 | 79.94 | 45.88 | 45.88 | 64.35 | 0.21 |
| Cocopeat:Rice husk:Vermicompost=50:30:20 | 171.35 | 143.73 | 212.99 | 198.35 | 158.05 | 77.00 | 27.62 | 69.26 | 54.62 | 14.32 | 54.94 | 54.94 | 71.35 | 40.30 | 40.30 | 52.34 | 0.19 |
| Cocopeat:Rice husk:Vermicompost=50:40:10 | 93.87 | 72.21 | 134.20 | 119.36 | 84.76 | 68.07 | 21.66 | 61.99 | 47.15 | 12.55 | 49.44 | 49.44 | 72.63 | 34.60 | 34.60 | 50.83 | 0.18 |
| Cocopeat:Rice husk:Vermicompost=60:20:20 | 91.49 | 68.57 | 133.70 | 121.71 | 80.76 | 63.64 | 22.92 | 65.13 | 53.14 | 12.19 | 52.94 | 52.94 | 83.19 | 40.95 | 40.95 | 64.35 | 0.19 |
| Soil:Cocopeat:Vermicompost=50:25:25 | 199.45 | 146.28 | 234.80 | 220.80 | 182.12 | 77.00 | 53.17 | 88.52 | 74.52 | 35.84 | 52.68 | 52.68 | 68.42 | 38.68 | 38.68 | 50.23 | 0.47 |

**Table S6**. Chemical properties of growing media/treatments (Expt. 2: Evaluation of growing media for vertical cultivation of capsicum and spinach)

| **Growing media/treatments** | **pH** | **EC** | **Moisture %** | **%N** | **%P** | **%K** | **%S** | **%OC** | **C/N** |
| --- | --- | --- | --- | --- | --- | --- | --- | --- | --- |
| Cocopeat=100% | 6.62 | 1.55 | 64.67 | 0.90 | 0.00 | 1.21 | 0.03 | 33.21 | 37.06 |
| Vermicompost=100% | 6.78 | 3.32 | 46.23 | 2.63 | 0.38 | 0.70 | 0.16 | 19.54 | 7.42 |
| Cocopeat:Rice husk=70:30 | 6.63 | 2.00 | 52.84 | 1.23 | 0.23 | 1.07 | 0.03 | 40.91 | 33.21 |
| Vermicompost:Rice husk=70:30 | 6.70 | 2.13 | 35.39 | 1.79 | 0.40 | 0.76 | 0.10 | 28.35 | 15.82 |
| Cocopeat:Rice husk:Vermicompost=30:20:50 | 7.29 | 1.66 | 47.57 | 1.79 | 0.28 | 0.87 | 0.09 | 29.16 | 16.27 |
| Cocopeat:Rice husk:Vermicompost=30:30:40 | 7.21 | 1.63 | 43.45 | 1.62 | 0.29 | 0.81 | 0.04 | 35.24 | 21.70 |
| Cocopeat:Rice husk:Vermicompost=30:40:30 | 6.20 | 2.19 | 36.61 | 1.68 | 0.21 | 0.87 | 0.04 | 37.67 | 22.42 |
| Cocopeat:Rice husk:Vermicompost=40:20:40 | 7.05 | 1.65 | 49.16 | 1.68 | 0.28 | 0.87 | 0.04 | 33.21 | 19.77 |
| Cocopeat:Rice husk:Vermicompost=40:30:30 | 6.49 | 1.87 | 44.75 | 1.68 | 0.32 | 0.86 | 0.06 | 35.24 | 20.98 |
| Cocopeat:Rice husk:Vermicompost=40:40:20 | 6.21 | 2.05 | 41.85 | 1.57 | 0.25 | 0.82 | 0.03 | 36.05 | 22.99 |
| Cocopeat:Rice husk:Vermicompost=50:20:30 | 6.76 | 1.78 | 52.56 | 1.51 | 0.18 | 0.86 | 0.04 | 31.59 | 20.89 |
| Cocopeat:Rice husk:Vermicompost=50:30:20 | 6.46 | 1.88 | 47.62 | 1.51 | 0.23 | 0.95 | 0.04 | 36.86 | 24.38 |
| Cocopeat:Rice husk:Vermicompost=50:40:10 | 6.13 | 2.13 | 42.40 | 1.68 | 0.24 | 0.84 | 0.02 | 35.64 | 21.21 |
| Cocopeat:Rice husk:Vermicompost=60:20:20 | 6.10 | 1.68 | 51.78 | 1.68 | 0.18 | 0.95 | 0.09 | 28.35 | 16.88 |
| Soil:Cocopeat:Vermicompost=50:25:25 | 6.60 | 1.84 | 34.63 | 0.89 | 0.15 | 0.63 | 0.04 | 10.17 | 11.43 |

**Table S7**. Raw data recorded for yield and yield attributes of capsicum and spinach (Expt. 2: Evaluation of growing media for vertical cultivation of capsicum and spinach)

| **Treatments** | **Treatment combination** | **Capsicum (no./pot)** | **Yield of capsicum (gm/pot)** | **Avg wt. (gm)** | **Avg wt. (gm)** | **Yield of capsicum (gm/pot)** | **Yield of capsicum (gm/pot)** | **(no./pot)** | **Yield of spinach (gm/pot)** | **Yield of spinach modified (gm/pot)** | **Yield of spinach (gm/pot)** | **No. of capsicum**  **(no./pot)** | **per capsicum wt.** | **Yield of capsicum (gm/pot)** |
| --- | --- | --- | --- | --- | --- | --- | --- | --- | --- | --- | --- | --- | --- | --- |
| Cocopeat=100% | T1R1 | 1.00 | 45.00 | 45.00 | 45.00 | 42.00 | 269.00 | 5.98 | 143.00 | 125.00 | 142.31 | 6.00 | 45.00 | 270.00 |
|  | T1R2 | 1.00 | 60.00 | 60.00 | 50.00 | 25.00 | 252.00 | 5.04 | 135.00 | 120.00 | 137.31 | 6.00 | 60.00 | 360.00 |
|  | T1R3 | 1.00 | 35.00 | 35.00 | 40.00 | 41.00 | 268.00 | 6.70 | 140.00 | 117.00 | 134.31 | 6.00 | 35.00 | 210.00 |
|  | **Mean** | **1.00** | **46.67** | **46.67** | **45.00** | **36.00** | **263.00** | **5.91** | **139.33** | **120.67** | **137.98** | **6.00** | **46.67** | **280.00** |
| Vermicompost=100% | T2R1 | 5.00 | 340.00 | 68.00 | 50.00 | 70.00 | 297.00 | 5.94 | 315.00 | 240.00 | 257.31 | 10.00 | 68.00 | 680.00 |
|  | T2R2 | 2.00 | 90.00 | 45.00 | 45.00 | 69.00 | 296.00 | 6.58 | 289.00 | 236.00 | 253.31 | 7.00 | 45.00 | 315.00 |
|  | T2R3 | 3.00 | 131.00 | 43.67 | 44.00 | 73.00 | 300.00 | 6.82 | 305.00 | 234.00 | 251.31 | 8.00 | 43.67 | 349.33 |
|  | **Mean** | **3.33** | **187.00** | **52.22** | **46.33** | **70.67** | **297.67** | **6.45** | **303.00** | **236.67** | **253.98** | **8.33** | **52.22** | **448.11** |
| Cocopeat:Rice husk=70:30 | T3R1 | 4.00 | 270.00 | 67.50 | 47.00 | 115.00 | 342.00 | 7.28 | 296.00 | 296.00 | 313.31 | 9.00 | 67.50 | 607.50 |
|  | T3R2 | 1.00 | 73.00 | 73.00 | 55.00 | 121.00 | 348.00 | 6.33 | 273.00 | 293.00 | 310.31 | 6.00 | 73.00 | 438.00 |
|  | T3R3 | 4.00 | 140.00 | 35.00 | 40.00 | 123.00 | 350.00 | 8.75 | 277.00 | 280.00 | 297.31 | 9.00 | 35.00 | 315.00 |
|  | **Mean** | **3.00** | **161.00** | **58.50** | **47.33** | **119.67** | **346.67** | **7.45** | **282.00** | **289.67** | **306.98** | **8.00** | **58.50** | **453.50** |
| Vermicompost:Rice husk=70:30 | T4R1 | 7.00 | 410.00 | 58.57 | 68.00 | 295.00 | 522.00 | 7.68 | 331.00 | 331.00 | 348.31 | 12.00 | 58.57 | 702.86 |
|  | T4R2 | 7.00 | 270.00 | 38.57 | 65.00 | 270.00 | 497.00 | 7.65 | 143.00 | 343.00 | 360.31 | 12.00 | 38.57 | 462.86 |
|  | T4R3 | 2.00 | 94.00 | 47.00 | 63.00 | 281.00 | 508.00 | 8.06 | 324.00 | 324.00 | 341.31 | 7.00 | 47.00 | 329.00 |
|  | **Mean** | **5.33** | **258.00** | **48.05** | **65.33** | **282.00** | **509.00** | **7.80** | **266.00** | **332.67** | **349.98** | **10.33** | **48.05** | **498.24** |
| Cocopeat:Rice husk:Vermicompost=30:20:50 | T5R1 | 3.00 | 120.00 | 40.00 | 55.00 | 143.00 | 370.00 | 6.73 | 320.00 | 316.00 | 333.31 | 8.00 | 40.00 | 320.00 |
|  | T5R2 | 3.00 | 140.00 | 46.67 | 57.00 | 140.00 | 367.00 | 6.44 | 297.00 | 305.00 | 322.31 | 8.00 | 46.67 | 373.33 |
|  | T5R3 | 3.00 | 126.00 | 42.00 | 54.00 | 139.00 | 366.00 | 6.78 | 313.00 | 313.00 | 330.31 | 8.00 | 42.00 | 336.00 |
|  | **Mean** | **3.00** | **128.67** | **42.89** | **55.33** | **140.67** | **367.67** | **6.65** | **310.00** | **311.33** | **328.64** | **8.00** | **42.89** | **343.11** |
| Cocopeat:Rice husk:Vermicompost=30:30:40 | T6R1 | 4.00 | 140.00 | 35.00 | 48.00 | 140.00 | 367.00 | 7.65 | 304.00 | 304.00 | 321.31 | 9.00 | 35.00 | 315.00 |
|  | T6R2 | 2.00 | 54.00 | 27.00 | 50.00 | 133.00 | 360.00 | 7.20 | 276.00 | 300.00 | 317.31 | 7.00 | 27.00 | 189.00 |
|  | T6R3 | 4.00 | 140.00 | 35.00 | 47.00 | 137.00 | 364.00 | 7.74 | 343.00 | 308.00 | 325.31 | 9.00 | 35.00 | 315.00 |
|  | **Mean** | **3.33** | **111.33** | **32.33** | **48.33** | **136.67** | **363.67** | **7.53** | **307.67** | **304.00** | **321.31** | **8.33** | **32.33** | **273.00** |
| Cocopeat:Rice husk:Vermicompost=30:40:30 | T7R1 | 2.00 | 90.00 | 45.00 | 52.00 | 122.00 | 349.00 | 6.71 | 375.00 | 300.00 | 317.31 | 7.00 | 45.00 | 315.00 |
|  | T7R2 | 3.00 | 114.00 | 38.00 | 48.00 | 124.00 | 351.00 | 7.31 | 318.00 | 298.00 | 315.31 | 8.00 | 38.00 | 304.00 |
|  | T7R3 | 5.00 | 170.00 | 34.00 | 52.00 | 121.00 | 348.00 | 6.69 | 309.00 | 295.00 | 312.31 | 10.00 | 34.00 | 340.00 |
|  | **Mean** | **3.33** | **124.67** | **39.00** | **50.67** | **122.33** | **349.33** | **6.91** | **334.00** | **297.67** | **314.98** | **8.33** | **39.00** | **319.67** |
| Cocopeat:Rice husk:Vermicompost=40:20:40 | T8R1 | 4.00 | 140.00 | 35.00 | 57.00 | 215.00 | 442.00 | 7.75 | 326.00 | 327.00 | 344.31 | 9.00 | 35.00 | 315.00 |
|  | T8R2 | 5.00 | 190.00 | 38.00 | 58.00 | 221.00 | 448.00 | 7.72 | 262.00 | 329.00 | 346.31 | 10.00 | 38.00 | 380.00 |
|  | T8R3 | 4.00 | 128.00 | 32.00 | 54.00 | 209.00 | 436.00 | 8.07 | 300.00 | 325.00 | 342.31 | 9.00 | 32.00 | 288.00 |
|  | **Mean** | **4.33** | **152.67** | **35.00** | **56.33** | **215.00** | **442.00** | **7.85** | **296.00** | **327.00** | **344.31** | **9.33** | **35.00** | **327.67** |
| Cocopeat:Rice husk: Vermicompost=40:30:30 | T9R1 | 1.00 | 47.00 | 47.00 | 57.00 | 169.00 | 396.00 | 6.95 | 370.00 | 318.00 | 335.31 | 6.00 | 47.00 | 282.00 |
|  | T9R2 | 2.00 | 76.00 | 38.00 | 58.00 | 186.00 | 413.00 | 7.12 | 291.00 | 310.00 | 327.31 | 7.00 | 38.00 | 266.00 |
|  | T9R3 | 6.00 | 177.00 | 29.50 | 55.00 | 177.00 | 404.00 | 7.35 | 328.00 | 324.00 | 341.31 | 11.00 | 29.50 | 324.50 |
|  | **Mean** | **3.00** | **100.00** | **38.17** | **56.67** | **177.33** | **404.33** | **7.14** | **329.67** | **317.33** | **334.64** | **8.00** | **38.17** | **290.83** |
| Cocopeat:Rice husk: Vermicompost=40:40:20 | T10R1 | 5.00 | 170.00 | 34.00 | 54.00 | 170.00 | 397.00 | 7.35 | 314.00 | 314.00 | 331.31 | 10.00 | 34.00 | 340.00 |
|  | T10R2 | 3.00 | 94.00 | 31.33 | 51.00 | 176.00 | 403.00 | 7.90 | 365.00 | 312.00 | 329.31 | 8.00 | 31.33 | 250.67 |
|  | T10R3 | 3.00 | 111.00 | 37.00 | 47.00 | 167.00 | 394.00 | 8.38 | 352.00 | 316.00 | 333.31 | 8.00 | 37.00 | 296.00 |
|  | **Mean** | **3.67** | **125.00** | **34.11** | **50.67** | **171.00** | **398.00** | **7.88** | **343.67** | **314.00** | **331.31** | **8.67** | **34.11** | **295.56** |
| Cocopeat:Rice husk:Vermicompost=50:20:30 | T11R1 | 4.00 | 137.00 | 34.25 | 48.00 | 96.00 | 323.00 | 6.73 | 329.00 | 268.00 | 285.31 | 9.00 | 34.25 | 308.25 |
|  | T11R2 | 4.00 | 130.00 | 32.50 | 46.00 | 97.00 | 324.00 | 7.04 | 319.00 | 250.00 | 267.31 | 9.00 | 32.50 | 292.50 |
|  | T11R3 | 2.00 | 96.00 | 48.00 | 49.00 | 94.00 | 321.00 | 6.55 | 298.00 | 249.00 | 266.31 | 7.00 | 48.00 | 336.00 |
|  | **Mean** | **3.33** | **121.00** | **38.25** | **47.67** | **95.67** | **322.67** | **6.77** | **315.33** | **255.67** | **272.98** | **8.33** | **38.25** | **312.25** |
| Cocopeat:Rice husk:Vermicompost=50:30:20 | T12R1 | 3.00 | 120.00 | 40.00 | 50.00 | 100.00 | 327.00 | 6.54 | 200.00 | 270.00 | 287.31 | 8.00 | 40.00 | 320.00 |
|  | T12R2 | 2.00 | 80.00 | 40.00 | 51.00 | 99.00 | 326.00 | 6.39 | 272.00 | 272.00 | 289.31 | 7.00 | 40.00 | 280.00 |
|  | T12R3 | 4.00 | 170.00 | 42.50 | 52.00 | 101.00 | 328.00 | 6.31 | 315.00 | 265.00 | 282.31 | 9.00 | 42.50 | 382.50 |
|  | **Mean** | **3.00** | **123.33** | **40.83** | **51.00** | **100.00** | **327.00** | **6.41** | **262.33** | **269.00** | **286.31** | **8.00** | **40.83** | **327.50** |
| Cocopeat:Rice husk:Vermicompost=50:40:10 | T13R1 | 4.00 | 140.00 | 35.00 | 45.00 | 110.00 | 337.00 | 7.49 | 169.00 | 280.00 | 297.31 | 9.00 | 35.00 | 315.00 |
|  | T13R2 | 1.00 | 54.00 | 54.00 | 54.00 | 108.00 | 335.00 | 6.20 | 308.00 | 279.00 | 296.31 | 6.00 | 54.00 | 324.00 |
|  | T13R3 | 3.00 | 165.00 | 55.00 | 55.00 | 106.00 | 333.00 | 6.05 | 349.00 | 261.00 | 278.31 | 8.00 | 55.00 | 440.00 |
|  | **Mean** | **2.67** | **119.67** | **48.00** | **51.33** | **108.00** | **335.00** | **6.58** | **275.33** | **273.33** | **290.64** | **7.67** | **48.00** | **359.67** |
| Cocopeat:Rice husk:Vermicompost=60:20:20 | T14R1 | 1.00 | 65.00 | 65.00 | 65.00 | 87.00 | 314.00 | 4.83 | 297.00 | 242.00 | 259.31 | 6.00 | 65.00 | 390.00 |
|  | T14R2 | 1.00 | 65.00 | 65.00 | 65.00 | 89.00 | 316.00 | 4.86 | 243.00 | 243.00 | 260.31 | 6.00 | 65.00 | 390.00 |
|  | T14R3 | 2.00 | 134.00 | 67.00 | 67.00 | 81.00 | 308.00 | 4.60 | 142.00 | 244.00 | 261.31 | 7.00 | 67.00 | 469.00 |
|  | **Mean** | **1.33** | **88.00** | **65.67** | **65.67** | **85.67** | **312.67** | **4.76** | **227.33** | **243.00** | **260.31** | **6.33** | **65.67** | **416.33** |
| Soil:Cocopeat:Vermicompost=50:25:25 | T15R1 | 1.00 | 65.00 | 65.00 | 65.00 | 65.00 | 292.00 | 4.49 | 314.00 | 142.00 | 159.31 | 6.00 | 65.00 | 390.00 |
|  | T15R2 | 1.00 | 65.00 | 65.00 | 65.00 | 68.00 | 295.00 | 4.54 | 292.00 | 135.00 | 152.31 | 6.00 | 65.00 | 390.00 |
|  | T15R3 | 1.00 | 67.00 | 67.00 | 67.00 | 64.00 | 291.00 | 4.34 | 282.00 | 132.00 | 149.31 | 6.00 | 67.00 | 402.00 |
|  | **Mean** | **1.00** | **65.67** | **65.67** | **65.67** | **65.67** | **292.67** | **4.46** | **296.00** | **136.33** | **153.64** | **6.00** | **65.67** | **394.00** |

**Table S8**. Raw data for vegetable yield and profitability under integrated and conventional farming systems (Expt. 3 Productivity and profitability of outdoor vertical farming integrated with conventional farming)

|  |  | **Yield from bed (kg/unit)** |  |  |  |  | **Yield from trellis (kg/unit)** |  |  | **Yield from vertically staked layer (kg/unit)** |  |  | **Yield on fence (**(kg/unit)**)** | **Total yield (**(kg/garden)**)** |
| --- | --- | --- | --- | --- | --- | --- | --- | --- | --- | --- | --- | --- | --- | --- |
|  |  | Tomato | Brinjal | Carrot | Broccoli | Total | Wax gourd | Red Amaranth | Total | Spinach | Capsicum | Total | Cowpea |  |
| Without vertically staked layer | Yield (kg) | 0 | 35 | 37 | 32.7 | 104.7 | 0 | 0 | 0 | 0 | 0 | 0 | 0 | 104.7 |
|  | Price (Tk.) |  | 40 | 40 | 50 |  |  |  | 0 |  |  | 0 |  |  |
|  | Gross return (Tk.) |  | 1400 | 1480 | 1635 | 4515 |  |  | 0 |  |  | 0 |  |  |
| With vertically staked layer | Yield (kg) | 29.83 | 11.5 | 20.98 | 13.04 | 75.35 | 27.3 | 20.18 | 47.48 | 12.86 | 15.99 | 28.85 | 4.2 | 307.56 |
|  | Price (Tk.) | 40 | 40 | 40 | 50 |  | 20 | 40 |  | 45 | 300 |  | 50 |  |
|  | Gross return (Tk.) | 1193.2 | 460 | 839.2 | 652 | 3144.4 | 546 | 807.2 | 1353.2 | 578.7 | 4797 | 5375.7 | 210 | 19956.6 |
|  |  |  |  |  |  |  |  |  |  |  |  |  |  |  |
|  |  |  |  |  |  |  |  |  |  |  |  |  |  |  |
|  |  |  |  |  |  |  |  |  |  |  |  |  |  |  |
|  |  |  |  |  |  |  |  |  |  |  |  |  |  |  |
|  |  |  |  |  |  |  |  |  |  |  |  |  |  |  |
|  |  |  |  |  |  |  |  |  |  |  |  |  |  |  |
|  |  |  |  |  |  |  |  |  |  | **cost** |  |  | with vertical layer | without |
|  | Broccoli | 16 no. | 35 tk/piece |  |  |  |  |  |  | bucket+media | 2475 |  | 495 |  |
|  |  | 16 | 35 | 560 | 56.852792 |  |  |  |  | bamboo | 1200 |  | 240 |  |
|  |  |  |  | 0 | 0 |  |  |  |  | labour (3 no.) | 1200 |  | 400 | 0 |
|  | Wax gourd | 16 no. | 30 tk/piece |  | 0 |  |  |  |  | Fertilizer |  |  | 250 | 250 |
|  |  | 16 | 30 | 480 | 19.834711 |  |  |  |  | Seed/seedling |  |  | 500 | 500 |
|  |  |  |  |  |  |  |  |  |  | Other |  |  | 200 | 200 |
|  |  |  |  |  |  |  |  |  |  | **Total** |  |  | **2085** | **950** |
